# SERS‐AI‐LUA‐Driven Salivary Diagnosis of Head and Neck Cancer Using Graphene‐Assisted Plasmonic Nanocorals

**DOI:** 10.1002/advs.202517710

**Published:** 2025-10-07

**Authors:** Hyo Jeong Seo, Boyou Heo, Jun‐Yeong Yang, Rowoon Park, Sung‐Gyu Park, Jiyoung Yeo, So Hee Park, Chan Kwon Jung, Min‐Young Lee, Jooin Bang, Jun‐Ook Park, Ho Sang Jung

**Affiliations:** ^1^ Advanced Bio and Healthcare Materials Research Division Korea Institute of Materials Science (KIMS) Changwon 51508 Republic of Korea; ^2^ Department of Medical Life Science College of Medicine The Catholic University of Korea Seoul 06591 Republic of Korea; ^3^ Department of Medical Sciences The Catholic University of Korea Seoul 06591 Republic of Korea; ^4^ Department of Hospital Pathology College of Medicine The Catholic University of Korea Seoul 06591 Republic of Korea; ^5^ Department of Otolaryngology‐Head and Neck Surgery Eunpyeong St. Mary's Hospital College of Medicine The Catholic University of Korea Seoul 03312 Republic of Korea; ^6^ Department of Otorhinolaryngology Seoul St. Mary's Hospital College of Medicine The Catholic University of Korea Seoul 06591 Republic of Korea; ^7^ School of Biomedical Engineering Korea University Seoul 02841 Republic of Korea

**Keywords:** head and neck cancer, label‐free diagnosis, machine learning, nonnegative least squares, plasmonic materials, salivary biomarkers, surface‐enhanced Raman scattering

## Abstract

The early detection of head and neck cancer (HNC) remains an important challenge owing to the lack of reliable noninvasive biomarkers. This study introduces a graphene‐assisted plasmonic nanocoral platform coupled with an artificial intelligence‐linear unmixing algorithm for diagnosing HNC from saliva and identifying associated metabolic biomarkers. The nanocoral structures, formed via a spontaneous gold growth mechanism on graphene templates, exhibit strong plasmonic enhancement and selective adsorption of volatile metabolites. Raman signals acquired from the saliva of HNC patients and healthy individuals are analyzed using a logistic regression model, achieving 98% classification accuracy. To identify potential metabolic biomarkers, candidate metabolites are initially selected based on spectral similarity using the Pearson correlation coefficient. Subsequently, the nonnegative least squares method is applied to refine this selection and extract the final set of biomarker candidates. This approach identifies 15 potential metabolic biomarkers, and their clinical relevance is corroborated through comparison with the findings of previous clinical studies. This study not only introduces a highly sensitive, noninvasive diagnostic platform for HNC but also establishes a robust framework for Raman‐based biomarker discovery, with potential applicability that warrants evaluation in other biofluid‐based disease models in future studies.

## Graphical abstract

The developed graphene‐assisted plasmonic nanocoral platform enables sensitive, label‐free surface‐enhanced Raman scattering analysis of salivary metabolites for head and neck cancer (HNC) detection. When combined with machine learning classification and nonnegative least squares‐based spectral deconvolution, this approach achieves 98% diagnostic accuracy in distinguishing HNC patients from healthy controls. Furthermore, the method identifies 15 potential biomarkers consistent with clinical findings, establishing a powerful framework for artificial intelligence‐integrated, noninvasive cancer diagnostics using saliva.

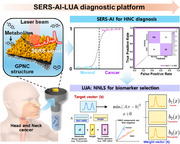

## 1 Introduction

Head and neck cancer (HNC) often manifests initially with minimal symptoms, leading to diagnostic delays until the disease progresses to an advanced stage when patients typically seek medical attention. Diagnostic delays also occur owing to difficulties in visualizing tumor locations via endoscopy or obtaining biopsies.^[^ ^1^, ^2^ ^]^ While promising, liquid biopsies currently lack well‐established biomarkers; the detection of circulating tumor DNA, circulating tumor cells, and exosomes is limited by their low concentrations, resulting in low sensitivity and specificity.^[^ ^3^, ^4^ ^]^ Consequently, most HNC diagnoses are made at advanced stages through imaging and biopsy, typically followed by surgical treatment. Furthermore, HNC substantially impacts the patient's quality of life due to its location. Postsurgical reconstruction frequently involves skin grafts, which can lead to long‐term discomfort and aesthetic concerns for patients.^[^ ^5^, ^6^ ^]^ Therefore, diagnostic technologies capable of detecting HNC in its earlier stages, particularly noninvasive methods suitable for the head and neck region, are urgently required. Saliva is an excellent biofluid source as it contains various molecules (metabolites, proteins, DNA, and RNA) and cells associated with HNC and can be collected noninvasively, making it an ideal liquid biopsy sample.^[^ ^7^, ^8^, ^9^ ^]^ Recent studies have analyzed DNA, RNA, and proteins in saliva for diagnostic applications, including the identification of human papillomavirus (HPV)‐driven cancers. Although several genetic markers, such as TP53 and PIK3CA mutations and HPV‐related markers, have been investigated for HNC diagnosis, limitations regarding their reliability persist.^[^ ^10^, ^11^ ^]^ Additionally, the diagnostic utility of specific mRNA or miRNA alterations (e.g., the downregulation of miR‐200a or miR‐125a observed in oral cancer) has not been validated across the diverse spectrum of HNC types.^[^ ^12^, ^13^ ^]^ Moreover, while salivary metabolomics has been employed to identify HNC‐associated metabolites, robust biomarker selection remains challenging. Thus, identifying and validating reliable biomarker candidates in saliva could establish new pathways for early HNC diagnosis.

Surface‐enhanced Raman scattering (SERS) technology amplifies the intrinsic Raman scattering signals of molecules and has been extensively researched for developing various label‐free detection sensors.^[^ ^14^, ^15^ ^]^ Recently, its application has expanded into the diagnostic field.^[^ ^16^ ^]^ The SERS effect arises when molecules adsorb onto or are near plasmonic nanomaterials, such as gold (Au) or silver, especially within nanogap structures that form “hot‐spot” regions. The intensified electromagnetic fields in these hot spots maximize the amplification of molecular Raman signals.^[^ ^17^ ^]^ Consequently, plasmonic nanomaterials facilitate the development of diverse label‐free SERS sensors. Coupled with artificial intelligence (AI) for analyzing complex biofluid signals, SERS offers potential as a novel diagnostic tool.^[^ ^18^, ^19^, ^20^ ^]^ However, many studies combining SERS and AI have primarily focused on classifying samples into patient versus healthy control groups. Most studies have emphasized identifying spectral features contributing to classification and assessing their statistical significance, suggesting their utility mainly for screening diagnostics. While valuable, identifying specific biomarkers alongside classification could aid in developing targeted treatment strategies. Currently, large‐scale untargeted screening, often using liquid chromatography‐mass spectrometry (LC‐MS/MS), is employed to discover new biomarkers.^[^ ^21^, ^22^ ^]^ However, analyzing numerous potential targets is challenging, and biologically elucidating their interconnections requires substantial time and effort. Therefore, an approach that appropriately filters potential biomarker targets identified by methods such as SERS is expected to enable more efficient biomarker discovery.

**Figure 1:**
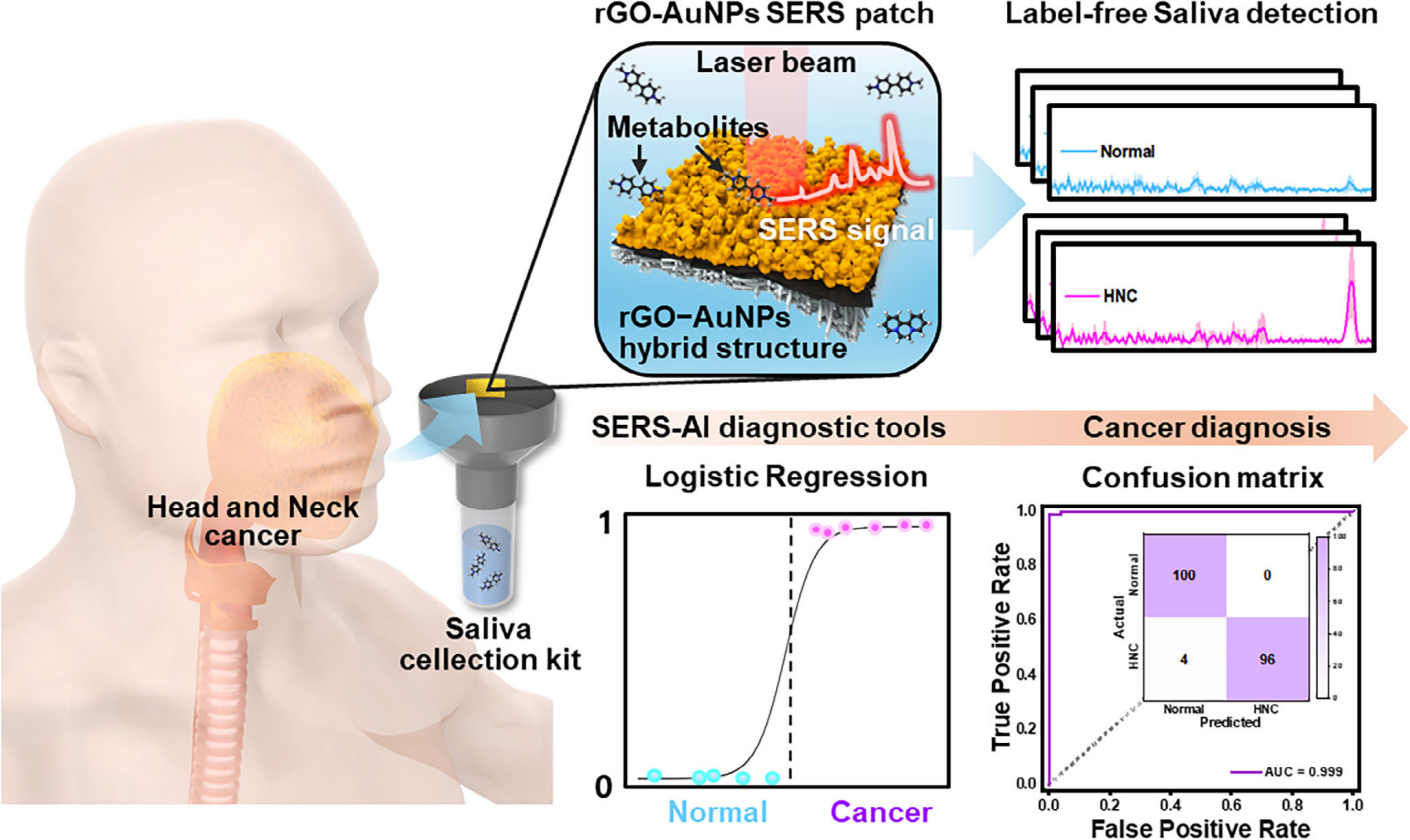
Schematic illustration of the AI‐assisted label‐free SERS sensor for HNC diagnosis.

In this study, label‐free Raman analysis was performed on saliva samples collected from patients with HNC and healthy individuals. **Figure** **1** illustrates the AI‐assisted label‐free SERS sensor developed for HNC diagnosis. Plasmonic substrates capable of effectively amplifying metabolic signals in saliva were fabricated by growing coral‐like Au nanostructures on a graphene template. The inherent wrinkles and defect sites on the graphene facilitated the initial growth of Au seeds without requiring an external reducing agent, and additional Au nanostructures were grown to prepare the nanocoral structure, which exhibited a strong plasmonic effect. Notably, the graphene component of the sensor material can capture volatile organic compounds through π–π stacking interactions, potentially enhancing the detection of volatile metabolites in saliva.^[^ ^23^, ^24^ ^]^ The developed graphene‐assisted plasmonic nanocoral (GPNC) substrate was then formulated into a diagnostic kit format and used to analyze saliva samples from HNC patients and healthy individuals. Using logistic regression (LR), a machine learning (ML) technique, the study successfully differentiated between the healthy and HNC groups with 98% classification accuracy. For biomarker screening, regions of the Raman spectra exhibiting substantial differences between the groups were identified. A reference Raman library was constructed for 39 metabolites known to be present in saliva. The potential contribution of each library metabolite to the patient spectra was initially assessed using the Pearson correlation coefficient (PCC) based on spectral similarity. Thereafter, the selected metabolites were relatively quantified using a linear unmixing algorithm (LUA) based on the nonnegative least squares (NNLS) method. Ultimately, 15 biomarker candidates, including thiocyanate, spermine, taurine, and putrescine, were identified in the saliva of HNC patients, which were consistent with previous reports. The materials developed in this study, combined with the Raman library‐based similarity analysis and the LUA‐NNLS method for biomarker quantification, demonstrate strong potential for noninvasive HNC diagnosis and may be extendable to other biofluid‐based disease models pending further validation.

## 2 Results and Discussion

### 2.1 Synthesis and Characterization of GPNC

**Figure 2:**
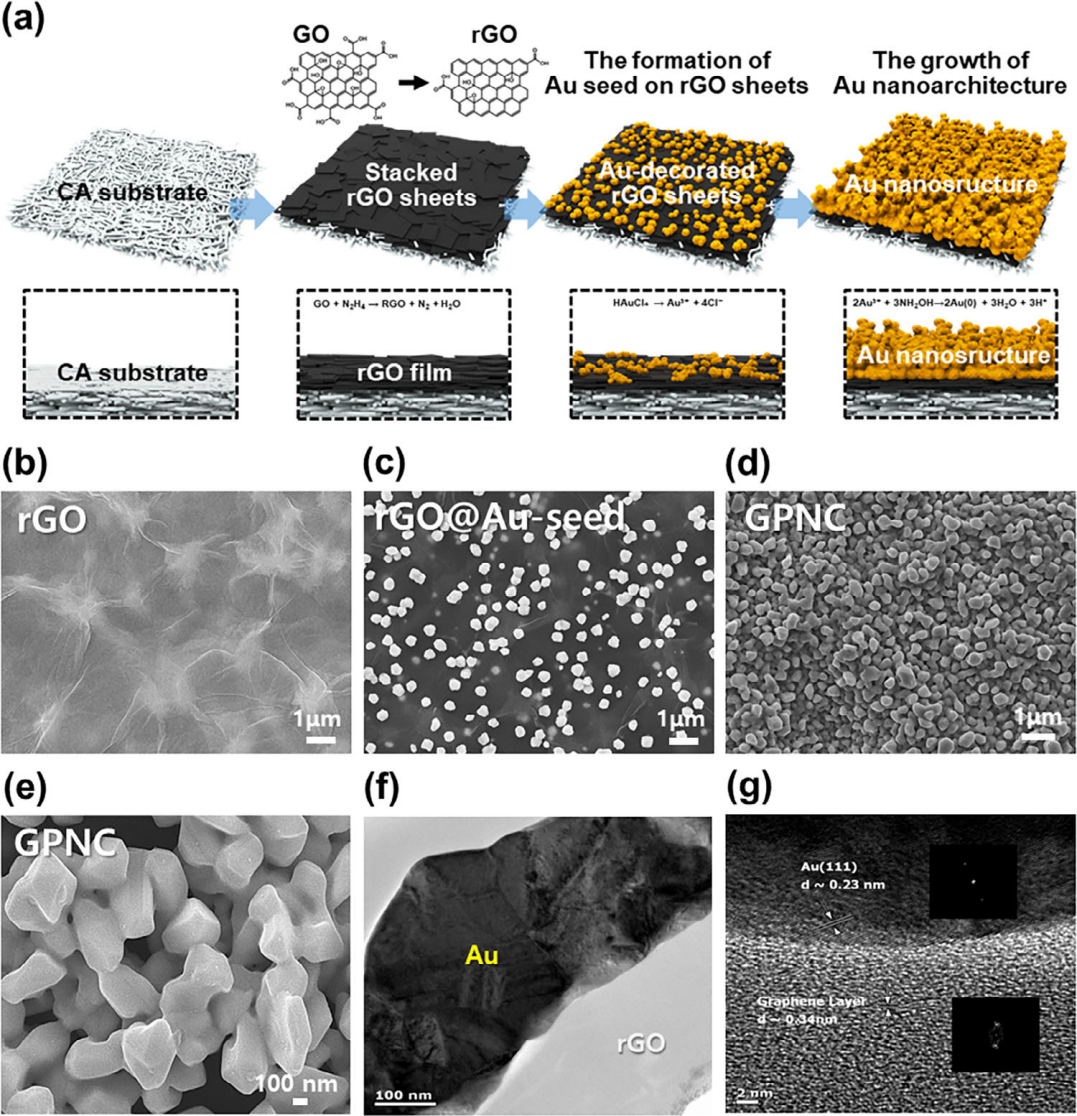
Materials characterization of the GPNC substrate. a) Schematic illustration of the GPNC formation mechanism, SEM images of b) rGO, c) rGO@Au‐seed, and d) GPNC (scale bar: 1 µm), e) higher magnification SEM image of GPNC (scale bar: 100 nm), f) cross‐sectional TEM image of GPNC showing the interface between Au and rGO layers, and g) HR‐TEM image of the GPNC structure.

As shown in **Figure** **2**a, the GPNC architecture was fabricated on flexible and porous cellulose acetate (CA) paper, selected for its ability to facilitate uniform solution dispersion across its surface.^[^ ^25^ ^]^ Initially, 3 mL of a 0.2 mg mL^−1^ graphene oxide (GO) solution was vacuum‐filtered onto the CA paper, followed by drying to adsorb graphene flakes onto the CA fiber surface. The surface‐coated GO was then reduced to reduced graphene oxide (rGO) using hydrazine vapor, followed by rinsing the surface thrice with deionized (DI) water to remove residual hydrazine. Scanning electron microscopy (SEM) images revealed a multi‐stacked and wrinkled rGO surface (Figure 2b; Figure S1a, Supporting Information). The uniform black color of the substrate confirmed complete coverage of the CA paper by the rGO layer. Following the successful preparation of the rGO surface, the substrate was immersed overnight in a 1 mm gold(III) chloride hydrate (HAuCl_4_) solution without any additional reducing agent. The substrate color changed to yellow, coinciding with the formation of polyhedral Au particles ≈200 nm in size (Figure 2c). Notably, these nanoparticles preferentially accumulated around the rGO wrinkles (Figure S1b, Supporting Information). This localization can be attributed to the diverse characteristics of the wrinkled regions. They possess high electron density, particularly from π electrons capable of spontaneously reducing Au^3+^ ions and structurally offer a large surface area with numerous defect sites, such as vacancies and functional groups, that act as nucleation points. Furthermore, charge transfer from the graphene facilitates electron donation, creating an environment conducive to Au^3+^ reduction. Subsequently, the rGO substrate decorated with these initial Au polyhedra was immersed in a 20 mm hydroxylamine hydrochloride (HH) solution. This step promoted further nanostructure growth on the graphene surface, increasing structural density seeded by the preformed nanoparticles. HH acts as a reducing agent, driving the autocatalytic reduction of Au^3^⁺ ions onto existing Au surfaces.^[^ ^26^, ^27^ ^]^ This process resulted in the formation of dense, partially interconnected nanocoral structures (Figure 2d; Figure S1c, Supporting Information). The interconnected morphology likely arises from additional Au, formed via the autocatalytic effect, growing along stable crystallographic directions and merging, ultimately forming the coral‐like architecture (Figure 2e). It is anticipated that the edge effects of single polyhedra particles can amplify the plasmonic effect, and the plasmonic coupling between adjacent nanostructures can maximize the Raman signal amplification.^[^ ^28^, ^29^ ^]^ The cross‐section of the prepared GPNC substrate was examined using transmission electron microscopy (TEM) after sample preparation via focused ion beam milling (Figure 2f). TEM analysis confirmed the well‐formed nanocoral structure residing on the rGO layer. Electron energy loss spectroscopy (EELS) mapping confirmed the elemental distribution, showing an rGO layer ≈7 nm thick beneath the Au nanostructures (Figure S2a,b, Supporting Information). High‐resolution TEM (HR‐TEM) revealed distinguishable phases at the rGO–Au interface but no substantial defects or voids, suggesting strong adhesion between the Au structures and the graphene surface (Figure 2g). Analysis of the diffraction pattern indicated that the rGO layer comprises both crystalline and amorphous phases. This is consistent with multi‐stacked GO undergoing reduction, where many C═C bonds reform graphitic domains (imparting crystallinity), while other regions remain as amorphous carbon.^[^ ^30^, ^31^ ^]^ To further analyze this, we conducted X‐ray diffraction (XRD) analysis on GO, rGO, the intermediate Au polyhedron‐decorated rGO, and the final GPNC substrate (Figure S3a, Supporting Information). The GO pattern showed a typical peak at 2*θ* = 10°−12° (001 reflection), corresponding to an interlayer spacing of 0.7–0.9 nm, indicative of oxygen functional groups, and a broad peak at 2*θ* = 23°−25° (002 reflection) associated with graphitic domains. The broadness of this peak suggested mixed crystalline and amorphous characteristics. In the rGO pattern, the oxide‐related (001) peak disappeared, while the (002) peak remained broad, consistent with incomplete reduction compared to pristine graphene. Both GO and rGO exhibited characteristic XRD patterns, with the resulting rGO displaying crystallinity alongside a defect‐rich nature. By contrast, when decorated with Au polyhedra, the rGO pattern was overlaid with peaks corresponding to the face‐centered cubic structure of Au (e.g., (111), (200), and (220)), confirming crystalline Au growth.^[^ ^32^ ^]^ For the final GPNC substrate, the intense signal from the thick Au nanocoral structure obscured the underlying rGO peaks, leaving only the Au diffraction pattern visible. This Au pattern indicated polycrystallinity (Figure S2c, Supporting Information), which, according to previous reports, can enhance SERS signals owing to additional light scattering at grain boundaries. The evolution of the samples during preparation was further monitored using Raman spectroscopy (Figure S3b, Supporting Information). Both GO and rGO displayed the characteristic D band (≈1305 cm^−1^) and G band (≈1580 cm^−1^).^[^ ^33^ ^]^ After the initial formation of polyhedral Au particles, both D and G bands were still observable owing to incomplete surface coverage. However, following the formation of the final GPNC structure using HH, only the G band remained visible. This suggests that the D band, associated with defects and oxygen functional groups, diminishes substantially during the HH reduction and GPNC formation process.

### 2.2 Plasmonic and Optical Properties of GPNC

**Figure 3:**
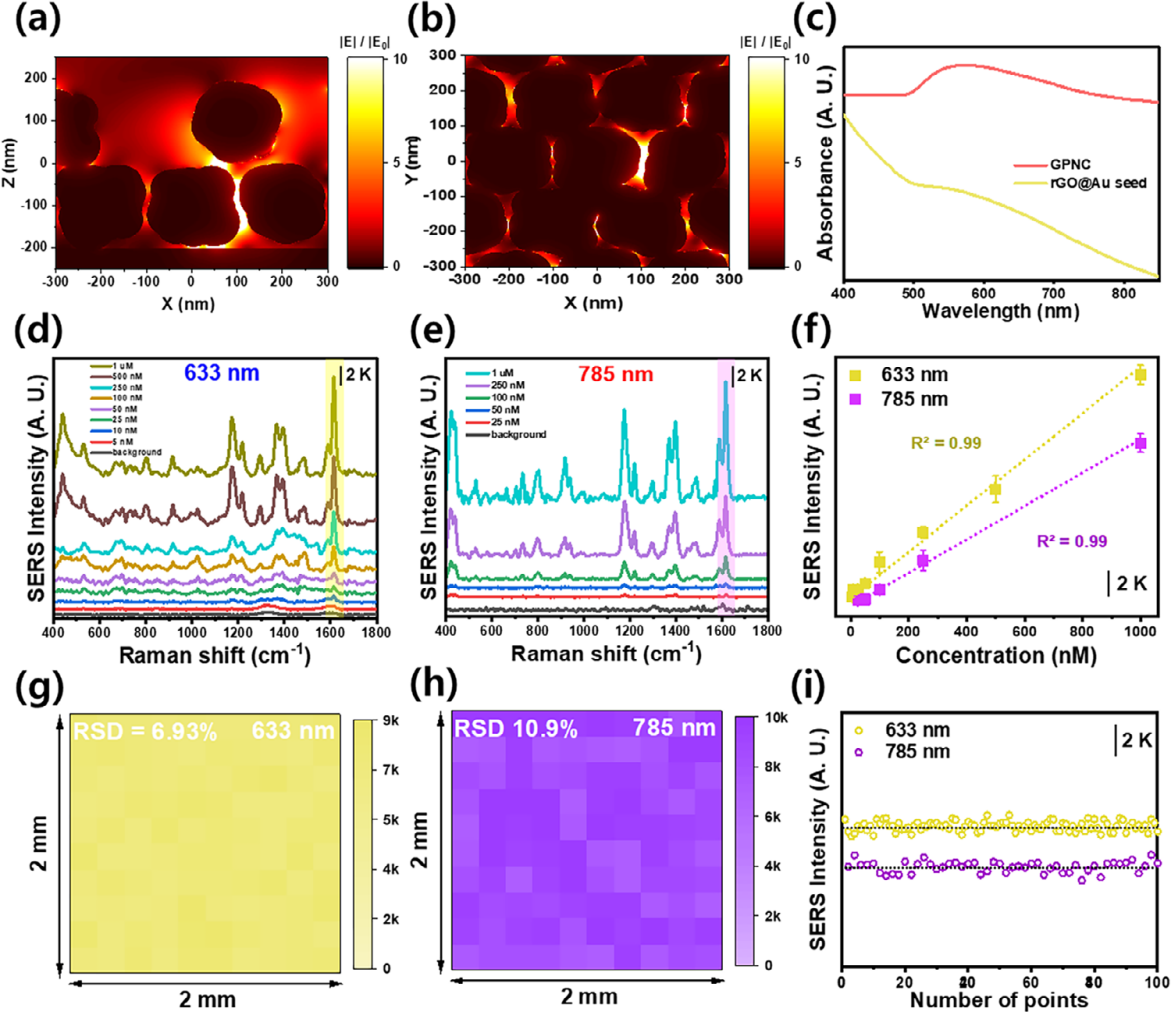
Plasmonic properties and SERS performance of the GPNC sensor. E‐field enhancement (|E|/|E_0_|) distributions from FDTD simulations: a) cross‐sectional view (X–Z plane) and b) top view (X–Y plane). c) UV–vis absorbance spectra of rGO@Au‐seed and GPNC, d) SERS spectra obtained using the GPNC sensor for various MG concentrations under 633 nm, and e) 785 nm excitation. f) calibration curves plotting SERS intensity versus MG concentration for both excitation wavelengths, g) Raman intensity maps of MG (1615 cm^−1^ peak) over a 2 × 2 mm^2^ area under 633 and h) 785 nm laser excitation, i) SERS signal intensities at 1615 cm^−1^ from the 100 points mapped in (g) and (h), demonstrating signal uniformity.

To investigate the electromagnetic field enhancement capabilities of the synthesized GPNC substrate, finite‐difference time‐domain (FDTD) simulations were conducted. As shown in **Figure** **3**a,b, dominant electric field (E‐field) amplification occurs primarily in the gaps between the constituent polyhedral nanoparticles of the GPNC structure, driven by edge effects. Figure S4 (Supporting Information) shows the E‐field distribution along the z‐axis based on a cross‐section, whereas Figure S5 (Supporting Information) shows the top view of the change along the y‐axis. The E‐field is formed both on the outermost layer and within the GPNC structure, indicating the formation of volumetric hotspots between the inner spaces of the nanostructures. This internal field enhancement suggests that molecules residing within these spaces can experience effective Raman signal amplification. The optical properties during GPNC synthesis were monitored using UV–vis absorbance spectroscopy (Figure 3c; Figure S6, Supporting Information). The GO precursor exhibited a characteristic absorbance peak at 230 nm (π → π* transition). After reduction to rGO, this peak shifted to ≈270 nm, corresponding to the n → π* transition.^[^ ^34^ ^]^ Following the initial deposition of Au polyhedral particles (rGO@Au‐seed stage), a relatively weak surface plasmon resonance (SPR) peak was detected around 520 nm. Upon complete synthesis of the GPNC structure, a broad SPR band centered around 570 nm was observed. This broadening and red‐shift are attributed to the interconnected nature and size distribution of the nanostructures within the GPNC.^[^ ^35^ ^]^ The SERS sensitivity of the GPNC substrate was evaluated using Malachite green (MG) as a Raman reporter dye across a range of concentrations, tested under two common excitation wavelengths: 633 and 785 nm. Before measurements, 10 µL aliquots of MG solutions were drop‐cast onto the substrate and allowed to dry (Figure 3d,e). Using 633 nm excitation, MG was detectable down to a concentration of 5 nm, with a calculated limit of detection (LOD) of 0.60 nm. Under 785 nm excitation, MG was detectable down to 25 nm, with an LOD of 3.18 nm. The Raman intensity showed a linear correlation with MG concentration under both wavelengths, indicating potential for molecular quantification (Figure 3f). However, considering the drop solution volume and the size of the substrate (0.5 cm × 0.5 cm), the LOD can be converted to a scale of pg cm^−^^2^, which is more feasible for interpreting actual quantities. Using the applied volume (10 µL), substrate area (0.25 cm^2^), and the molecular weight of MG, the LODs can be converted to 8.76 pg cm^−^^2^ (at 633 nm) and 46.44 pg cm^−^^2^ (at 785 nm), confirming the high sensitivity of the SERS substrate. By contrast, signal uniformity is crucial for developing reliable sensors. Raman mapping was performed across a 2 mm × 2 mm area (100 points) using both 633 and 785 nm excitation. The resulting relative standard deviation (RSD) values were 6.93% (633 nm) and 10.90% (785 nm), indicating good signal homogeneity (Figure 3g,h). Furthermore, the intensity of a representative MG peak at 1615 cm^−1^ exhibited minimal variation across the 100 randomly measured points (Figure 3i) indicating reliable signal generation from the developed substrate. Spectral uniformity was also confirmed by comparing spectra obtained from 20 random positions under both wavelengths (Figure S7a,b, Supporting Information). To evaluate reproducibility, batch‐to‐batch variation was assessed for GPNC. Ten independent batches were fabricated and tested with 1 µm MG under identical conditions. The GPNC substrates showed an average RSD of 7.47%, indicating that the platform offers superior reproducibility (Figure S8, Supporting Information). This high degree of uniformity and reproducibility can be attributed to the dense distribution of hotspots within the laser illumination area. SEM images (Figure S1c, Supporting Information) show that more than five polyhedral nanoparticles, which constitute the nanocoral architecture, are present within a 1 µm^2^ area. Given the excitation laser spot diameter of 22 µm, ≈1900 such particles are interrogated simultaneously in a single measurement. As the measured SERS signal represents an ensemble average from all illuminated plasmonic particles within the spot, the GPNC substrate provides a highly uniform platform for SERS‐based molecular analysis.

### 2.3 AI‐Assisted HNC Diagnosis using Saliva

**Figure 4:**
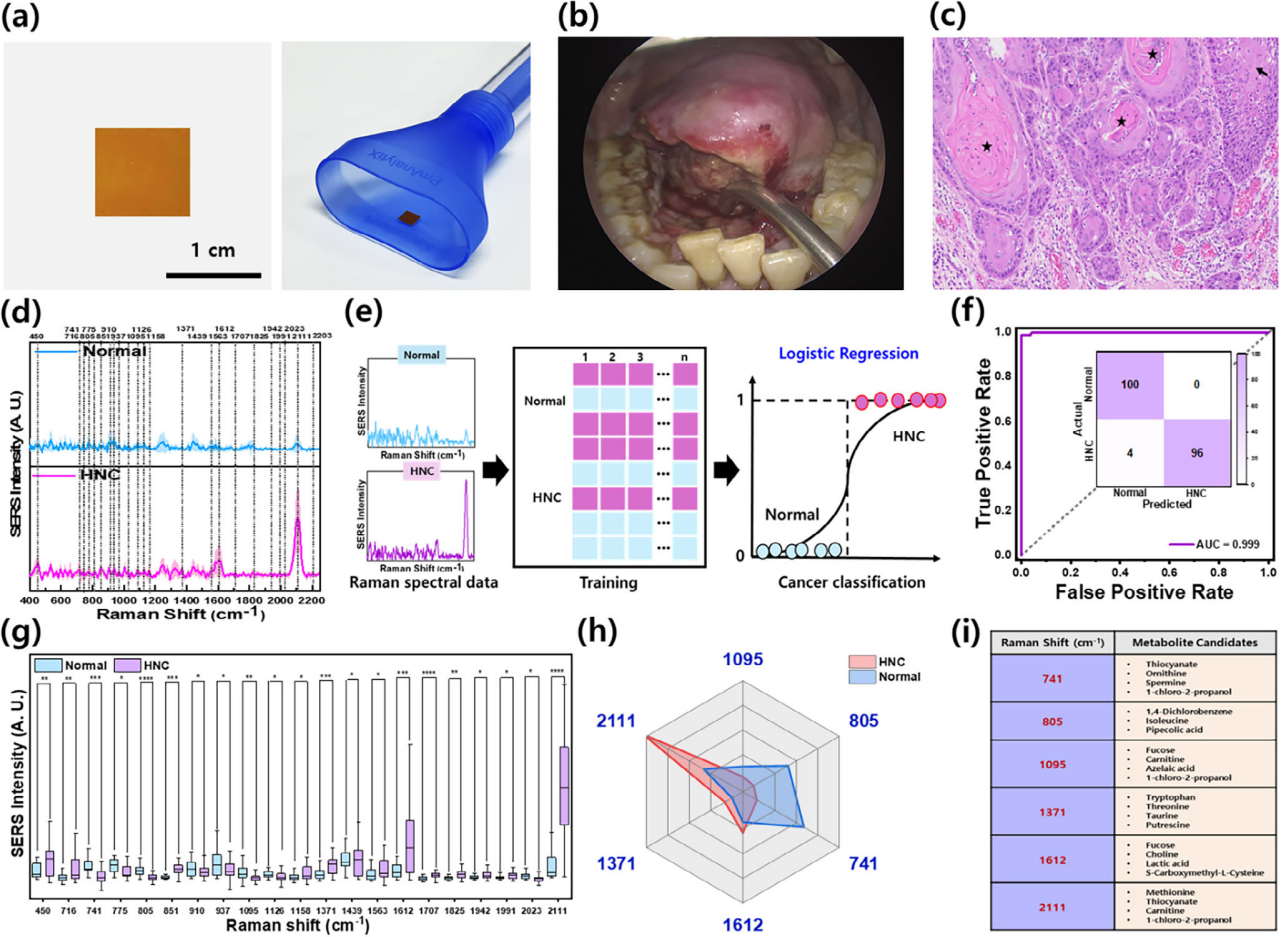
SERS analysis of human saliva samples for HNC diagnosis. a) Photograph of the GPNC substrate integrated into a commercial saliva collection kit funnel, b) intraoral photograph showing squamous cell carcinoma on the tongue of an HNC patient, c) hematoxylin and eosin staining of squamous cell carcinoma of the tongue, showing tumor nests with keratin pearls (stars), dyskeratotic cells, and intercellular bridges (arrow) within a fibrotic stroma (×20), d) average SERS spectra of saliva supernatant from healthy control (Normal) and HNC patients, e) schematic illustration of the LR‐assisted ML model used for classification, f) ROC curve and confusion matrix (inset) for the LR model classification performance, g) comparison of mean SERS intensities (± standard error) between normal and HNC groups at statistically discriminative Raman shifts (*p < 0.05, **p < 0.01, ***p < 0.001, and ****p < 0.0001; *t*‐test), h) radar plot visualizing the average intensities at the six most significantly different Raman shift positions for normal and HNC groups, i) table listing potential metabolite candidates corresponding to the selected discriminative Raman peaks shown in (h).

Following thorough characterization, the GPNC substrate was adapted into a prototype saliva collection kit (**Figure** **4**a; Figure S9, Supporting Information). A 1 cm^2^ section of the GPNC material, prepared on a 4.5 cm diameter CA substrate, was attached to the funnel surface of a standard commercial saliva collection tube. This study aimed to differentiate between HNC patients and healthy control individuals based on salivary SERS spectra. Saliva samples were collected according to a strict protocol: participants fasted for over 12 h (no food or drink, including water) prior to providing their first morning saliva sample. For HNC patients, samples were obtained immediately before their initial surgery. These procedures minimized variability related to diet, circadian rhythm, and treatment, thereby reducing inter‐individual differences in the salivary metabolome. A representative laryngoscopy photograph shows squamous cell carcinoma on the tongue (Figure 4b). All patient samples underwent histological analysis (Figure 4c), and inclusion required confirmation of HNC diagnosis by a physician. Immediately after collection, all saliva samples were frozen before analysis; samples were thawed and centrifuged to remove cell debris and other impurities using only the supernatant. For SERS measurements, a fixed volume of 10 µL saliva supernatant was applied to the GPNC substrate. Spectra were subsequently normalized to the intensity of the urea peak at ≈1000 cm^−1^, a band often associated with oral pH regulation.^[^ ^36^ ^]^ Urea is a major salivary component that remains relatively stable across individuals and therefore provides a reliable internal reference. This normalization minimizes technical variability arising from differences in salivary pH and ionic strength, ensuring that statistical comparisons reflect true biological differences rather than technical artifacts.

A total of 50 saliva samples were analyzed, comprising 25 from HNC patients with tumors in diverse anatomical subsites (tonsil, tongue, larynx, hypopharynx, retromolar trigone (RMT), pharynx, oropharynx, and lymphoma) and 25 from healthy individuals with no diagnosed disease codes (Figures S10 and S11, Supporting Information). All analyses were conducted on this age‐balanced cohort of clinically confirmed cases and healthy controls. SERS spectra were acquired using 785 nm laser excitation (4 mW power, 7 s integration time). The 785 nm wavelength was chosen to minimize potential background interference from the rGO component of the substrate, which could obscure weak analyte signals near the characteristic peaks of rGO if 633 nm excitation was used (Figure S12, Supporting Information). As shown in Figure 4d, compared to the normal group, a distinct peak intensity difference can be observed at 2111 cm^−1^ for the HNC group, with moderate variations in other spectral regions. Statistically significant differences across the spectral range were identified using *t*‐tests (indicated by the dotted line in Figure 4d). Before investigating the differences in Raman peak positions, a ML model based on LR was applied to classify the samples as HNC or healthy control (Figure 4e). Leveraging the spectral data, particularly the prominent difference at 2111 cm^−1^, the LR model achieved accurate classification, yielding 100% specificity and 96% sensitivity, as demonstrated by the confusion matrix and receiver operating characteristic (ROC) curve in Figure 4f. Despite the presence of a dominant peak intensity difference at a specific Raman shift position, an LR approach was employed instead of dimensionality reduction techniques such as principal component analysis or partial least squares‐discriminant analysis.^[^ ^37^ ^]^ On the held‐out test set (Figure 4f), the LR model achieved 98% accuracy with an area under the curve (AUC) of 0.999. Stratified fivefold cross‐validation further yielded consistent results, with a mean accuracy of 93.4 ± 1.2% and a mean AUC of 0.95 ± 0.01 (Figure S13a,b, Supporting Information). The held‐out test set performance reflects the result from a single split of the cohort, while the cross‐validation analysis provides a more stable estimate averaged across multiple splits. The consistency between these two evaluation strategies supports the robustness of the model performance. This decision was motivated by the fact that such linear regression methods reduce dimensionality by generating new composite features, potentially obscuring the contribution of individual Raman shifts critical for interpreting individual spectral characteristics.^[^ ^38^ ^]^ Even when spectral data exhibit clear differences, utilizing the original features directly can be more informative. LR offers a substantial advantage in this context, as it efficiently handles high‐dimensional data without requiring prior dimensionality reduction. Furthermore, LR incorporates regularization to mitigate the risk of overfitting, making it well‐suited for this analysis.^[^ ^39^ ^]^ Following successful classification, a detailed investigation was conducted to identify specific Raman shifts exhibiting statistically significant intensity differences between the groups (*p* < 0.05, *t*‐test), which were identified and systematically sorted (Figure 4g). The primary goal was to associate these substantial spectral features with specific salivary components, particularly metabolites, to identify potential biomarkers. To facilitate this, a reference library was created by measuring the SERS spectra of 70 candidate metabolites (selected based on literature reports of salivary components in healthy and HNC patients) on the GPNC substrate (Figure S14 and Table S1, Supporting Information).^[^ ^40^, ^41^, ^42^, ^43^, ^44^, ^45^, ^46^, ^47^, ^48^, ^49^, ^50^, ^51^, ^52^, ^53^, ^54^, ^55^, ^56^, ^57^, ^58^, ^59^, ^60^, ^61^, ^62^, ^63^, ^64^ ^]^ Importantly, metabolites were not identified by a single peak but rather by their entire spectral profile. Reproducible SERS spectra were obtained for 39 of the 70 candidates, which formed the working library for subsequent full‐spectrum analyses. As shown in Figure S14 (Supporting Information), usable Raman spectra were obtained for 39 of these metabolites, and their characteristic peak positions were cataloged for comparison against the statistically significant peaks observed in the saliva spectra (Figure 4g). Subsequently, the six Raman shifts showing the most substantial differences between the HNC and normal groups were selected for further visualization using a radar plot (Figure 4h). Potential metabolite candidates corresponding to these six key Raman shifts are listed in Figure 4i, representing potential biomarkers that appear upregulated or downregulated in the saliva of HNC patients in this cohort. Peak intensities were interpreted only as semi‐quantitative indicators under uniform sampling and measurement conditions, and absolute concentrations were not inferred. Classification was performed using the full spectra, while metabolite screening applied PCC only as a pre‐screening step followed by NNLS full‐spectrum unmixing to evaluate multi‐band contributions. A more detailed analysis involving mathematical models for biomarker selection and discussion of their clinical relevance is presented in Section 2.5.

### 2.4 Salivary Metabolite Capture Test using GPNC Substrate

**Figure 5:**
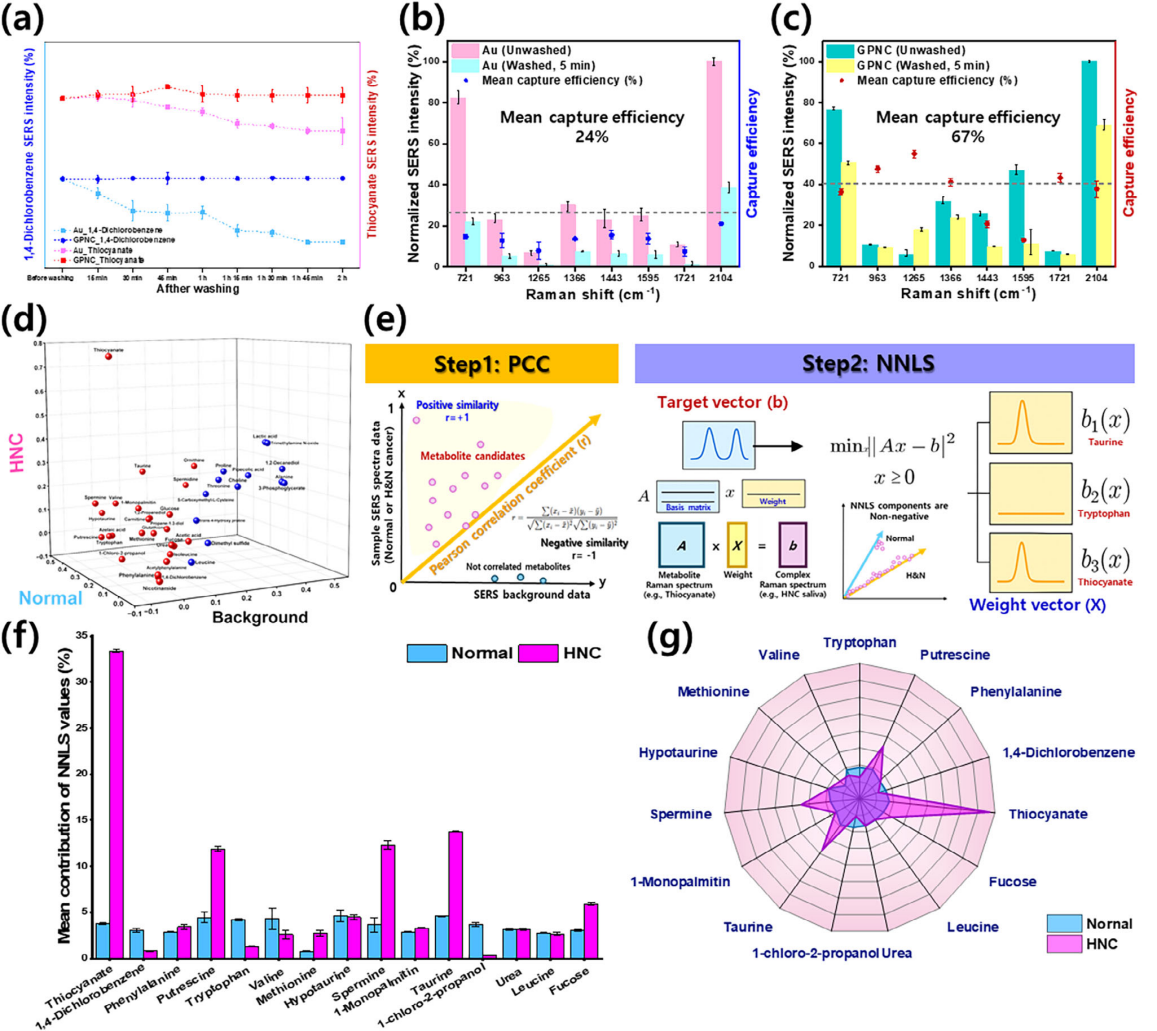
Mathematical model and relative quantification of metabolites using combined PCC and NNLS analysis. a) Time‐dependent SERS monitoring of salivary volatile metabolite standards (1,4‐dichlorobenzene and thiocyanate comparing signal stability on GPNC versus control Au substrates, b) comparison of capture efficiency on Au substrates, both unwashed and washed (5 min), c) comparison on GPNC substrates under the same conditions, d) 3D visualization of PCC analysis, mapping reference library metabolites based on spectral similarity to averaged Normal (healthy control), HNC patient, and background SERS signals, e) schematic workflow illustrating the combined PCC–NNLS algorithm applied to SERS spectra, f) comparison of NNLS‐derived relative contributions for the top 15 differentiating metabolites between Normal and HNC saliva samples, g) radar plot visualizing the NNLS‐based relative contributions of selected metabolites from f) for the Normal and HNC groups.

The rGO component of the developed GPNC substrate is known to exhibit intermolecular interactions with various molecules via π–π and hydrophobic interactions involving its graphitic domains. Given that some salivary metabolites are volatile, the presence of rGO within the GPNC structure was anticipated to enhance the retention of these substances, thereby potentially improving SERS sensing performance. Among the salivary metabolite candidates identified in Figure 4i, the volatile compounds 1,4‐dichlorobenzene and thiocyanate were selected to investigate the capture ability of the GPNC substrate. Interestingly, as shown in **Figure** **5**a, the GPNC substrate maintained stable SERS signals for these two components for up to 2 h. By contrast, a control Au substrate synthesized without an rGO layer showed a continuous signal decrease over the same period. This enhanced retention on GPNC is attributed to molecular capture through π–π interactions between the delocalized π electrons of the 1,4‐dichlorobenzene benzene ring and the C≡N triple bond of thiocyanate^−^.^[^ ^65^, ^66^, ^67^, ^68^ ^]^ In the case of GPNC, although the rGO layer is beneath the Au surface, molecules can adsorb and generate signals at the Au interface. Moreover, after adsorption, the presence of rGO decelerates the evaporation of substances compared to the substrates without rGO, thereby maintaining the signal intensity over time. The stability of the GPNC substrate was further evaluated by subjecting it to five sequential DI water washes followed by 100‐point Raman mapping after each cycle. The mean peak intensities remained stable with no significant decay across washes, with RSD values of 7.01%, 7.04%, 8.69%, 8.04%, and 7.42% after 1×, 2×, 3×, 4×, and 5× washing, respectively (Figure S15a, Supporting Information). SEM images acquired before and after 5x washing additionally confirmed that the nanoscale tip sharpness and interparticle gaps were preserved without structural collapse (Figure S15b, Supporting Information). These results demonstrate that the GPNC substrate maintains both morphological and spectral stability under repeated washing conditions.

Prior to the additional adsorption experiments, standard calibration curves were first established using thiocyanate (0.1–1 mm) and 1,4‐dichlorobenzene (0.1–1 mm) (Figure S16b,d, Supporting Information). The UV–vis absorbance increased linearly with concentration across the tested range, and linear regression analysis yielded coefficients of determination (R^2^ > 0.99), confirming excellent linearity (Figure S16a,c, Supporting Information). These calibration curves were subsequently applied to calculate the residual concentrations (Ct) in the supernatant after adsorption. To directly validate the adsorption process beyond SERS intensity, depletion experiments were performed by incubating GPNC, Au, and CA paper substrates with thiocyanate and 1,4‐dichlorobenzene solutions at an initial concentration (C_0_) of 0.2 mm for 2 h. After incubation, the supernatant solutions were collected and analyzed by UV–vis spectroscopy. In the case of 0.2 mm solutions, a reduction of Ct compared with the initial *C*_0_ was observed, providing evidence of analyte adsorption by the substrates. The decrease in absorbance was most pronounced for the GPNC substrate, indicating the highest adsorption efficiency, followed by the Au substrate and CA paper. Control samples without substrate retained their original absorbance (Figure S16e,f, Supporting Information). These results provide direct evidence that analytes are effectively adsorbed onto the rGO–Au hybrid surface, thereby corroborating the adsorption mechanism deduced from SERS measurements. In addition, the adsorption of analytes onto the rGO layer can be rationalized by well‐established non‐covalent interactions. The extended π‐conjugated network of graphene enables strong π–π stacking with aromatic compounds, while its hydrophobic basal plane favors adsorption of non‐polar analytes through hydrophobic interactions. Charged or polar molecules (e.g., thiocyanate) can further engage in electrostatic or cation–π interactions with the electron‐rich rGO surface. Notably, non‐polar aromatic compounds such as 1,4‐dichlorobenzene can adsorb effectively via π–π stacking between the benzene ring and the graphene lattice, as well as hydrophobic interactions with the basal plane. These combined interactions explain the efficient adsorption observed on the GPNC substrate and are consistent with prior reports on graphene‐based adsorption mechanisms.^[^ ^69^, ^70^, ^71^, ^72^ ^]^ Subsequently, the retention of Raman signals from complex biological samples was investigated using human saliva on both GPNC and control Au substrates without rGO. Saliva samples were applied to both substrate types, dried, and then washed with DI water for 5 min. SERS spectra acquired before and after washing were compared (Figure S17, Supporting Information). The results qualitatively indicated that overall signal retention across the entire spectrum was substantially enhanced by the presence of the rGO layer in the GPNC substrate. To quantify this effect, eight representative peak positions were identified, and the intensities before and after washing were calculated (Figure 5b,c). This intensity ratio was defined as the capture efficiency. The control Au substrate retained an average of only 24% of the initial signal intensity after washing, whereas the GPNC substrate exhibited a substantially higher average capture efficiency of ≈67%. This demonstrates that the rGO component effectively enhances the retention of salivary metabolites, leading to more robust and stable SERS signals suitable for analysis.

### 2.5 Combined PCC and NNLS for Salivary HNC Biomarker Selection

Building upon the metabolite capture ability demonstrated by the substrate (Section 2.4), which suggested the GPNC platform could acquire meaningful SERS signals from saliva, a method for semi‐quantitative analysis was developed to evaluate the contributions of specific metabolites to the observed spectra. While Figure 4d–i highlighted potential biomarker candidates based on substantial spectral differences, this section details a refined selection process using combined computational methods. A reference SERS library of 39 salivary metabolites potentially associated with HNC or found in healthy saliva was constructed (Figure S14 and Table S1, Supporting Information, as mentioned above). From this library, metabolites substantially contributing to the observed spectral differences between the HNC patient and healthy control saliva samples were initially screened using the PCC. The PCC was calculated by comparing each metabolite spectrum with background signals in a binary manner: normal versus background and HNC versus background. A PCC value approaching +1 or −1 indicates a strong linear relationship (high spectral similarity) between the reference metabolite and saliva‐sample spectra,^[^ ^73^ ^]^ whereas a value near 0 implies low similarity. A schematic workflow illustrating the combined PCC–NNLS algorithm applied to the SERS spectra is shown in Figure 5e. As shown in Figure S18 (Supporting Information), metabolites exhibiting higher similarity (positioned farther from the central dotted line toward either the normal or HNC axis) were considered more substantial contributors. These sorted metabolites were visualized in three dimensions, as shown in Figure 5d. Based on this PCC analysis, 25 metabolites (1‐chloro‐2‐propanol, 1‐monopalmitin, 1,2‐propanediol, 1,4‐dichlorobenzene, acetic acid, acetylphenylalanine, azelaic acid, carnitine, fucose, glucose, glutamine, hypotaurine, isoleucine, methionine, nicotinamide, ornithine, phenylalanine, propane‐1,3‐diol, putrescine, spermidine, spermine, taurine, thiocyanate, urea, and valine), marked with red circles in Figure 5d, were selected as potential biomarker candidates capable of distinguishing between the patient and control groups.

Subsequently, the relative contributions, or secretion levels, of these selected biomarker candidates within the saliva spectra were estimated using the NNLS method. NNLS is a LUA technique used to approximate an observed vector as a weighted linear combination of reference vectors, constrained such that all weights are nonnegative.^[^ ^74^, ^75^ ^]^ This approach enabled the deconvolution of the complex observed saliva spectra into a weighted sum of the preselected metabolite reference spectra, facilitating a semi‐quantitative comparison of metabolite distributions between the normal and HNC groups. Among the 25 candidates identified via PCC analysis (Figure 5d), the top 15 metabolites showing the most important NNLS‐derived contributions differentiating the groups were selected and visualized in Figure 5f,g to highlight group‐specific secretion patterns. The calculated relative contributions were normalized to the contribution of urea, chosen owing to its role in salivary pH regulation and relatively similar levels in both cancer patients and healthy individuals because of its minimal dependency on the presence or absence of the disease. The NNLS analysis revealed substantially elevated levels of thiocyanate, phenylalanine, putrescine, methionine, spermine, 1‐monopalmitin, taurine, and fucose in HNC patient saliva than the normal group. thiocyanate, a major salivary component and substrate for salivary peroxidase,^[^ ^76^ ^]^ may be elevated owing to cancer‐associated inflammation and oxidative stress; it is produced during cyanide detoxification by rhodanese and acts as a substrate for myeloperoxidase, generating hypothiocyanous acid involved in redox signaling and stress responses within the tumor microenvironment.^[^ ^77^, ^78^ ^]^ Phenylalanine, an essential amino acid and precursor of tyrosine and catecholamines, shows altered accumulation in HNC patients owing to enhanced amino acid metabolism supporting protein synthesis and cell proliferation.^[^ ^79^ ^]^ Putrescine and spermine elevation reflects upregulation of the polyamine biosynthetic pathway (arginine → ornithine → putrescine → spermidine → spermine) required for rapid cell proliferation.^[^ ^80^, ^81^ ^]^ Methionine, vital for cell growth, DNA synthesis, and methylation regulation, exhibits abnormal activation—termed methionine dependency—observed in various cancers, including HNC.^[^ ^82^ ^]^ Elevated 1‐monopalmitin in the saliva of HNC patients likely indicates altered lipid metabolism associated with tumor growth. HNC cells exhibit enhanced de novo lipogenesis and membrane remodeling, which can increase production of monoacylglycerols such as 1‐monopalmitin.^[^ ^83^ ^]^ Taurine, a sulfur‐containing amino acid with antioxidant and anti‐inflammatory properties, is upregulated in HNC, likely as a compensatory response to oxidative stress and inflammation in the tumor microenvironment.^[^ ^83^, ^84^ ^]^ Fucose, involved in glycoprotein fucosylation, is involved in cell adhesion and immune recognition. Elevated fucose in HNC may reflect aberrant glycosylation that promotes tumor progression and immune evasion.^[^ ^85^ ^]^ Although most salivary metabolites discussed in this study have been previously reported, the consistency between their relative secretion levels and those reported previously supports the effectiveness of the proposed LUA method, NNLS.

Conversely, the NNLS analysis indicated reduced relative contributions of 1,4‐dichlorobenzene, tryptophan, valine, hypotaurine, 1‐chloro‐2‐propanol, and leucine in HNC saliva samples. Decreased 1,4‐dichlorobenzene could relate to impaired gland function, altered detoxification, or metabolic reprogramming in the tumor microenvironment.^[^ ^84^ ^]^ Tryptophan reduction is often linked to its increased catabolism via the kynurenine pathway in HNC, supporting NAD⁺ synthesis, immune suppression, and oxidative stress resistance.^[^ ^86^ ^]^ Lower levels of the branched‐chain amino acids valine and leucine, essential for protein synthesis and energy metabolism, may reflect increased consumption by rapidly proliferating tumor cells.^[^ ^84^, ^87^ ^]^ Hypotaurine depletion might occur despite pathway upregulation owing to its rapid conversion to taurine under oxidative stress in the HNC microenvironment.^[^ ^84^ ^]^ In addition, 1‐chloro‐2‐propanol emerged as a candidate metabolite of interest. While its biological mechanism remains to be elucidated, this compound has previously been reported in the saliva of HNC/OSCC patients and included in salivary biomarker panels in subsequent reviews.^[^ ^45^, ^88^ ^]^ In the present study, however, the trend observed for 1‐chloro‐2‐propanol did not fully align with findings reported in the literature, which may reflect characteristics specific to the patient group analyzed. Further studies with larger and independent cohorts will be required to clarify its clinical relevance. Overall, the integrated PCC–NNLS approach enabled a comprehensive characterization of salivary metabolic alterations, highlighting the potential of Raman spectroscopy as a noninvasive diagnostic tool for HNC. This study successfully applied AI (LR for classification) and LUA‐based computational methods (PCC screening, NNLS unmixing) to SERS spectra from clinical saliva samples acquired using the GPNC platform. Potential biomarkers were identified, and their relative abundance patterns were found to be largely consistent with those reported in existing metabolomic studies of HNC, underscoring the robustness of this approach. The integration of this methodology with plasmonic materials holds promise for noninvasive diagnostics in HNC and may be extendable to other biofluid‐based disease contexts pending further validation. Furthermore, expanding the reference Raman library could facilitate the discovery of novel metabolic biomarkers for diseases where they are currently lacking.^[^ ^89^, ^90^ ^]^ The cohort size of this study was relatively modest (n = 50), and validation in larger, multi‐center cohorts will be necessary to confirm the broader clinical applicability of this approach.

## 3 Conclusion

In this study, we developed a noninvasive diagnostic platform integrating plasmonic GPNC‐based SERS sensors with an AI‐assisted spectral analysis framework. This framework employs a combination of the PCC for initial screening and NNLS, a LUA method, for classifying healthy individuals versus HNC patients and identifying potential metabolic biomarkers. The GPNC substrate, fabricated via spontaneous Au growth on graphene templates, demonstrated strong plasmonic enhancement and effective retention of salivary analytes, including volatile compounds. Utilizing ML‐based classification (LR) combined with the spectral deconvolution approach (PCC–NNLS), the platform achieved 98% classification accuracy in distinguishing HNC patients from healthy controls. Furthermore, analysis of a constructed salivary metabolite Raman library using the PCC–NNLS workflow led to the identification of 15 potential biomarkers (including thiocyanate, taurine, and ornithine), whose differential abundance between the groups aligns with existing clinical literature. Overall, this work highlights the substantial potential of integrating advanced SERS substrates with AI‐driven data analysis, specifically incorporating LUA tools, for noninvasive, label‐free disease diagnostics and biomarker discovery. The proposed GPNC‐SERS platform shows promise for the effective and rapid diagnosis of HNC using saliva. Moreover, it may also be extendable in future studies to other biofluid‐based disease models, provided that additional validation is performed.

## 4 Experimental Section

### Materials

GO (5 mg mL^−1^ solution), hydrazine hydrate, HH, HAuCl_4_, MG, methionine, leucine, valine, acetylphenylalanine, 3‐phosphoglycerate, 1,2‐decanediol, trans‐4‐hydroxyproline, S‐carboxymethyl‐L‐cysteine, 1‐monopalmitin, 1,4‐dichlorobenzene, urea, trimethylamine N‐oxide, phenylalanine, glucose, choline, acetic acid, spermidine, tryptophan, threonine, potassium thiocyanate, taurine, spermine, putrescine, propane‐1,3‐diol, proline, pipecolic acid, ornithine, nicotinamide, lactic acid, hypotaurine, glutathione, fucose, dimethyl sulfide, carnitine, azelaic acid, alanine, 1‐chloro‐2‐propanol, and 1,2‐propanediol were purchased from Sigma–Aldrich (St Louis, MO, USA). CA membrane filters (pore size: 0.45 µm) were purchased from Hyundai Micro Co., LTD (Seoul, South Korea). Saliva samples from healthy individuals were collected at the Korea Institute of Materials Science (KIMS, Changwon, South Korea; institutional review board (IRB) No. P01‐202310‐02‐005) and Seoul St. Mary's Hospital (Seoul, South Korea; IRB No. PC24TNSE0171). Saliva samples from HNC patients were obtained from Seoul St. Mary's Hospital under the same IRB approval (IRB No. PC24TNSE0171). All samples were collected in accordance with relevant ethical guidelines and regulations.

### Fabrication of the GPNC Sensor

A stock solution of GO (5 mg mL^−1^) was diluted with DI water to a final concentration of 0.2 mg mL^−1^. Subsequently, 3 mL of this diluted GO solution (0.2 mg mL^−1^) was vacuum‐filtered onto the CA membrane filter and allowed to dry. The GO‐coated substrate was then chemically reduced to rGO by exposure to hydrazine hydrate vapor overnight and subsequently rinsed with DI water to remove residual reactants. For Au seed deposition, the prepared rGO/CA substrate was immersed in a HAuCl_4_ solution (1 mm) overnight without any additional reducing agent. Finally, to grow the nanocoral structure, the Au‐seeded substrate was immersed overnight in a freshly prepared aqueous solution containing 10 mm HAuCl_4_ and 20 mm HH.

### Material Characterization

The surface morphology and nanostructure of the GPNC were examined using FE‐SEM (JSM‐6700F, JEOL).TEM (JEM‐2200FS equipped with an Image Cs‐corrector, JEOL) was used to analyze the internal structure and crystallinity. Elemental mapping was performed using EELS integrated with the TEM system. Crystal structure analysis was conducted using XRD with a Panalytical X'Pert Pro diffractometer.

### FDTD Simulations

3D FDTD simulations were performed using commercial software (Lumerical, Ansys; version 2021 R1.2) to model the E‐field distribution and enhancement characteristics of the GPNC structure. The geometrical parameters for the model were derived from representative SEM and TEM images. The GPNC model comprises Au spherical nanoparticles(diameters ranging from 200 to 210 nm) uniformly distributed on an rGO layer (7 nm thick). The interior of the Au structure was filled with randomly overlapped rGO flakes. A plane wave with x‐polarization and a wavelength of 785 nm was normally incident upon the GPNC surface. A uniform mesh size of 0.5 nm was employed. The frequency‐dependent dielectric functions of Au and rGO at 785 nm were set as ε_Au = −21.6448 + 0.7433i and ε_rGO = 2.863 + 1.913i, respectively. The background refractive index was set to 1. Periodic boundary conditions were applied in the x‐ and y‐directions, while perfectly matched layers were used in the +z direction and a perfect electric conductor boundary was used in the −z direction.

### SERS Activity Test of the GPNC Sensor

UV–vis absorbance spectroscopy was used to evaluate the SERS performance. The rGO@Au‐seed and final GPNC layers were transferred from the CA paper substrates onto transparent adhesive films, which were then placed at the bottom of a 96‐well plate for optical signal collection using a microplate reader (SpectraMax M2, Molecular Devices, USA). UV–vis spectra of GO and rGO dispersions were measured using a quartz cuvette. To determine the SERS LOD, 10 µL aliquots of MG solutions at various concentrations (ranging from 1 µm to 5 nm) were drop‐cast onto the GPNC substrate and allowed to dry completely. Raman spectra were then acquired using a portable Raman spectrometer (NS220, Nanoscope Systems, Daejeon, South Korea) under two different laser excitation conditions: 633 nm laser (1.5 mW power, 3 s exposure) and 785 nm (4 mW power, 7 s exposure). The LOD was calculated using the formula LOD = 3σ S^−1^, where σ represents the standard deviation of the background signal intensity at the target Raman peak measured from blank samples (n = 3), and S is the slope derived from the LR of the calibration curve (intensity vs concentration). Signal uniformity was assessed by acquiring Raman spectra of MG (1 µm, 10 µL) from 100 randomly selected points across the substrate surface using the same respective laser conditions.

### Reproducibility Test of the GPNC Substrate

Reproducibility across different batches was assessed by fabricating ten independent GPNC substrates and measuring the Raman intensities of 1 µm MG under identical conditions. The RSD of the characteristic peak intensities was calculated to evaluate batch‐to‐batch reproducibility.

### Salivary Metabolite Capture Test

To evaluate the ability of the GPNC substrate to retain volatile substances, 10 µL of 10 mm 1,4‐dichlorobenzene and thiocyanate solutions were drop‐cast onto the GPNC surface, and SERS signals were monitored at 15‐min intervals over 2 h using the NS220 Raman spectrometer (Nanoscope Systems, South Korea, 785 nm excitation, 4 mW power, 7 s exposure). Additionally, the retention of Raman signals was tested using human saliva supernatant applied to both GPNC and control Au substrates (lacking rGO). After saliva application and drying, the substrates were rinsed with DI water for 5 min. SERS spectra were measured before and after washing under the same Raman conditions to evaluate analyte retention efficiency.

### Stability Test of GPNC Substrates

To evaluate the stability of the GPNC substrates under repeated washing, each substrate was sequentially rinsed five times with DI water. After each washing cycle, Raman spectra of MG (1 µm, 10 µL) were acquired from 100 randomly selected points using the same measurement conditions as described above (785 nm excitation, 4 mW, 2 s). The RSD values of the characteristic Raman peak intensities were calculated to assess spectral stability. In addition, substrates were examined by SEM before and after the 5x washing cycles to evaluate morphological integrity, focusing on nanoscale tip sharpness and interparticle gaps.

### Additional Adsorption Tests

To further quantify analyte uptake on different substrates, UV–vis calibration and adsorption experiments were performed. Standard solutions of thiocyanate (0.1, 0.2, 0.5, and 1 mm) and 1,4‐dichlorobenzene (0.1, 0.2, 0.5, and 1 mm) were prepared. Aliquots of 200 µL from each solution were dispensed into a 96‐well quartz plate, and UV–vis absorbance spectra were recorded to generate calibration curves. These curves were subsequently used to determine residual concentrations in adsorption experiments. Substrates, including GPNC, Au, and CA paper, were cut to cover the bottom of 5 mL glass vials and immersed in 1 mL of a 0.2 mm solution. The mixtures were incubated statically for 2 h at room temperature. After incubation, 200 µL aliquots of the supernatant were transferred into a 96‐well quartz plate, and the Ct were quantified by UV–vis spectroscopy. A decrease in supernatant absorbance compared with the initial control solution was interpreted as higher adsorption efficiency of the corresponding substrate.

### SERS Measurement of Saliva

For analysis, a 10 µL aliquot of each saliva supernatant sample was drop‐cast onto the GPNC substrate and allowed to air dry completely. SERS spectra were acquired using the NS220 portable Raman spectrometer with 785 nm laser excitation (4 mW power, 7 s exposure time). Each saliva sample was measured five times at different positions on the substrate to account for spatial variability. Additionally, the SERS spectra of individual metabolite candidates were acquired by drop‐casting 10 µL of their respective 10 mm aqueous solutions onto separate GPNC substrates. All saliva samples were centrifuged to separate sputum and debris, and the supernatant was used for analysis.

### ML Analysis

For model training and evaluation, the cohort of 50 saliva spectra (25 HNC and 25 healthy) was randomly divided into a 70% training set and a 30% held‐out test set using stratified sampling to preserve class balance. LR with L2‐regularization, as implemented in scikit‐learn (v1.2.2), was applied. To mitigate potential overfitting and obtain more robust performance estimates, stratified fivefold cross‐validation was additionally performed on the training data. Model performance was reported as accuracy, sensitivity, specificity, and area under the ROC curve (AUC), averaged across folds. This workflow ensured that all preprocessing and evaluation steps were performed in a leakage‐free manner and that performance metrics reflected generalizable classification ability.

### Combined PCC–NNLS Calculation

Figure 5e illustrates the combined PCC–NNLS algorithm that was applied to the SERS spectra. First, PCC analysis was used to distinguish reference metabolites whose spectra exhibited high similarity to background signals from those specifically correlated with the spectral features of either the HNC or normal group. This analysis used a custom Python code to process SERS spectra acquired from normal, HNC, background, and individual metabolites measured using the GPNC sensor. All PCC calculations were performed within the 400–2300 cm^−1^ spectral range. Specifically, correlation coefficients were calculated between the SERS spectrum of each reference metabolite and the averaged SERS spectra corresponding to the background, normal, and HNC groups, allowing for the identification of metabolites highly correlated with background signals or specifically associated with either normal or HNC samples. To quantify the contribution of individual metabolites, an NNLS analysis was conducted using only the normal and HNC groups. Prior to this analysis, all metabolite spectra were min‐max normalized based on their highest peak, and the relative Raman cross‐sections were considered in the analysis. The NNLS computation was performed using scipy.optimize package in Python, with the spectral range set from 400 to 2300 cm^−1^. This computation determined the proportion of each metabolite contributing to the normal and HNC spectra. To estimate uncertainty (error bars) of the NNLS results, the analysis was repeated five times employing slightly varied sub‐ranges within the 400–2300 cm^−1^ window. For computational consistency, all SERS spectra were normalized within a consistent range using min‐max normalization.

NNLS solves the following equation:

(1) Ax≈bsubjecttox≥0

where A is the basis matrix composed of reference spectra (each column representing a metabolite), b is the target vector representing the observed Raman spectrum of a sample, and x is the nonnegative weight vector indicating the contributions of each metabolite. NNLS allows the estimation of the relative abundance of known components within a complex biological sample, enabling semi‐quantitative analysis. This approach allowed for the profiling of metabolite distributions in the normal and HNC groups, thereby highlighting group‐specific metabolic signatures. In this study, a metabolite was operationally defined as a low‐molecular‐weight compound previously reported in saliva that produced reproducible SERS spectra on the GPNC substrate within 400–2300 cm^−1^.

## Conflict of Interest

The authors declare no conflict of interest.

## Supporting information



Supporting Information

## Data Availability

The data that support the findings of this study are available from the corresponding author upon reasonable request.
