# Supplementary material for: SERS‐AI‐LUA‐Driven Salivary Diagnosis of Head and Neck Cancer Using Graphene‐Assisted Plasmonic Nanocorals
Source: Adv Sci (Weinh). 2025 Oct 7;12(48):e17710. doi: 10.1002/advs.202517710 (PMC12752647; doi:10.1002/advs.202517710)
Supplement: Supplementary file 1 — Supporting Information [file ADVS-12-e17710-s001.docx]

Supporting Information

**SERS-AI-LUA-Driven Salivary Diagnosis of Head and Neck Cancer Using Graphene-Assisted Plasmonic Nanocorals**

*Hyo Jeong Seo, Boyou Heo, Jun-Yeong Yang, Rowoon Park,* *Sung-Gyu Park, Jiyoung Yeo, So Hee Park, Chan Kwon Jung, Min-Young Lee, Jooin Bang, Jun-Ook Park* and Ho Sang Jung^*^*

H. J. Seo, B. Heo, Dr. J.-Y. Yang, Dr. R. Park, Dr. S.-G. Park, Dr. M.-Y. Lee

Advanced Bio and Healthcare Materials Research Division, Korea Institute of Materials Science (KIMS), Changwon 51508, Republic of Korea

Prof. J. Bang

Department of Otolaryngology-Head and Neck Surgery, Eunpyeong St. Mary’s Hospital,

College of Medicine, The Catholic University of Korea, Seoul 03312, Republic of Korea

Prof. J.-O. Park*

Department of Otorhinolaryngology, Seoul St. Mary’s Hospital,

College of Medicine, The Catholic University of Korea, Seoul 06591, Republic of Korea

Dr. J. Yeo

Department of Medical Life Science, College of Medicine, The Catholic University of Korea, Seoul 06591, Republic of Korea

S. H. Park

Department of Medical Sciences, The Catholic University of Korea, Seoul 06591, Republic of Korea

Prof. C. K. Jung

Department of Hospital Pathology, College of Medicine, The Catholic University of Korea, Seoul 06591, Republic of Korea

Prof. H. S. Jung*

School of Biomedical Engineering, Korea University, Seoul 02841, Republic of Korea

*Corresponding Authors E-mail: [junook2000@catholic.ac.kr](mailto:junook2000@catholic.ac.kr); [jhs0626@korea.ac.kr](mailto:jhs0626@korea.ac.kr)

Keywords: plasmonic materials, label-free diagnosis, surface-enhanced Raman scattering, head and neck cancer, salivary biomarkers, machine learning, nonnegative least squares


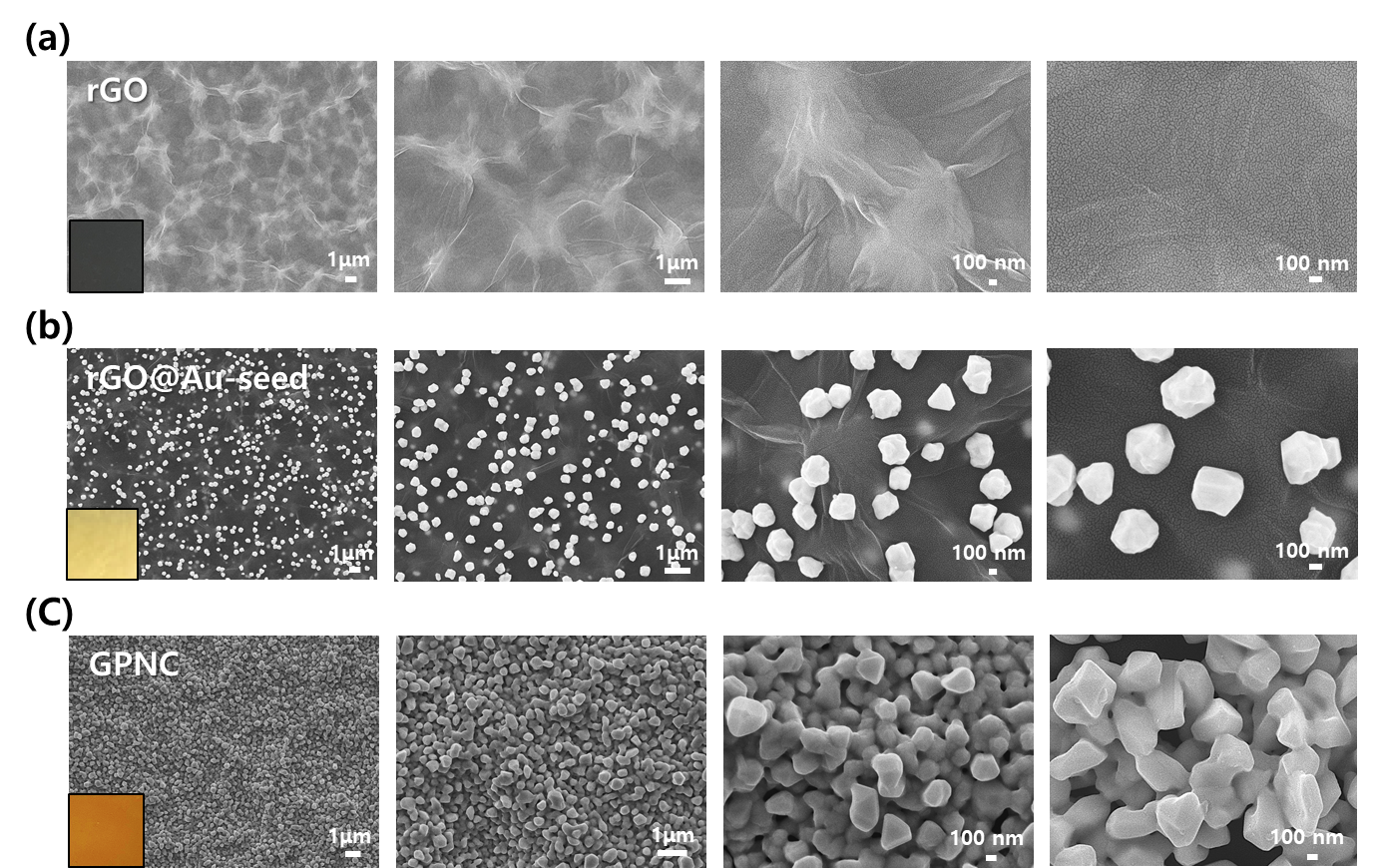
**Figure S1**. SEM images of (a) rGO, (b) rGO@Au-seed, and (c) GPNC at various magnifications.


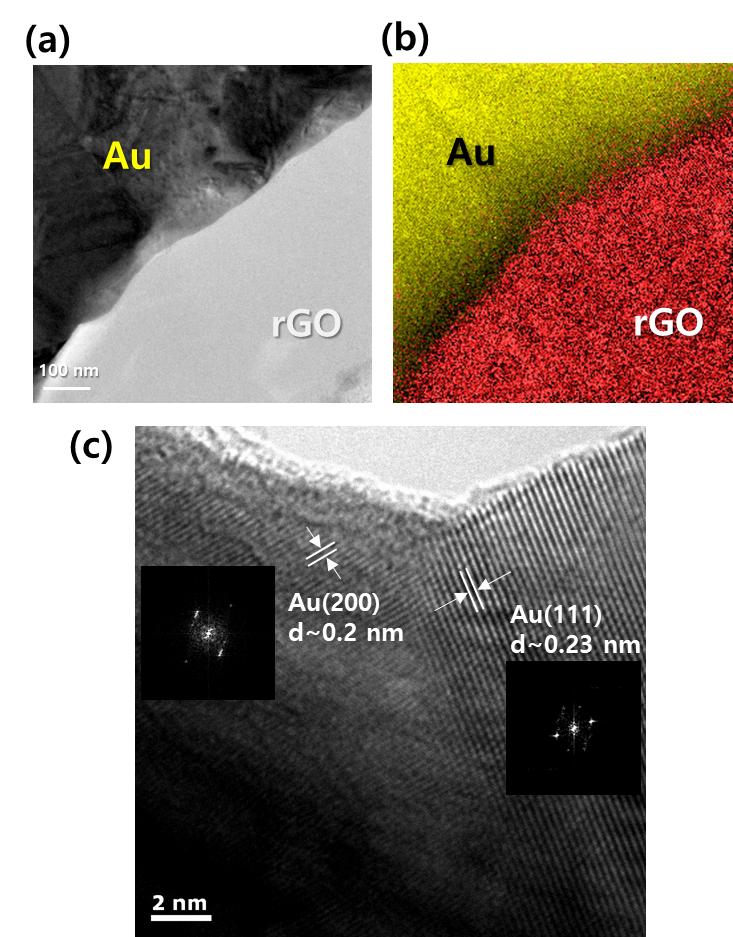


**Figure S2**. (a) TEM images of GPNC structure, (b) EELS image of Au (yellow) and rGO (red) elements of the GPNC, (c) HR-TEM images of the GPNC architecture.

**
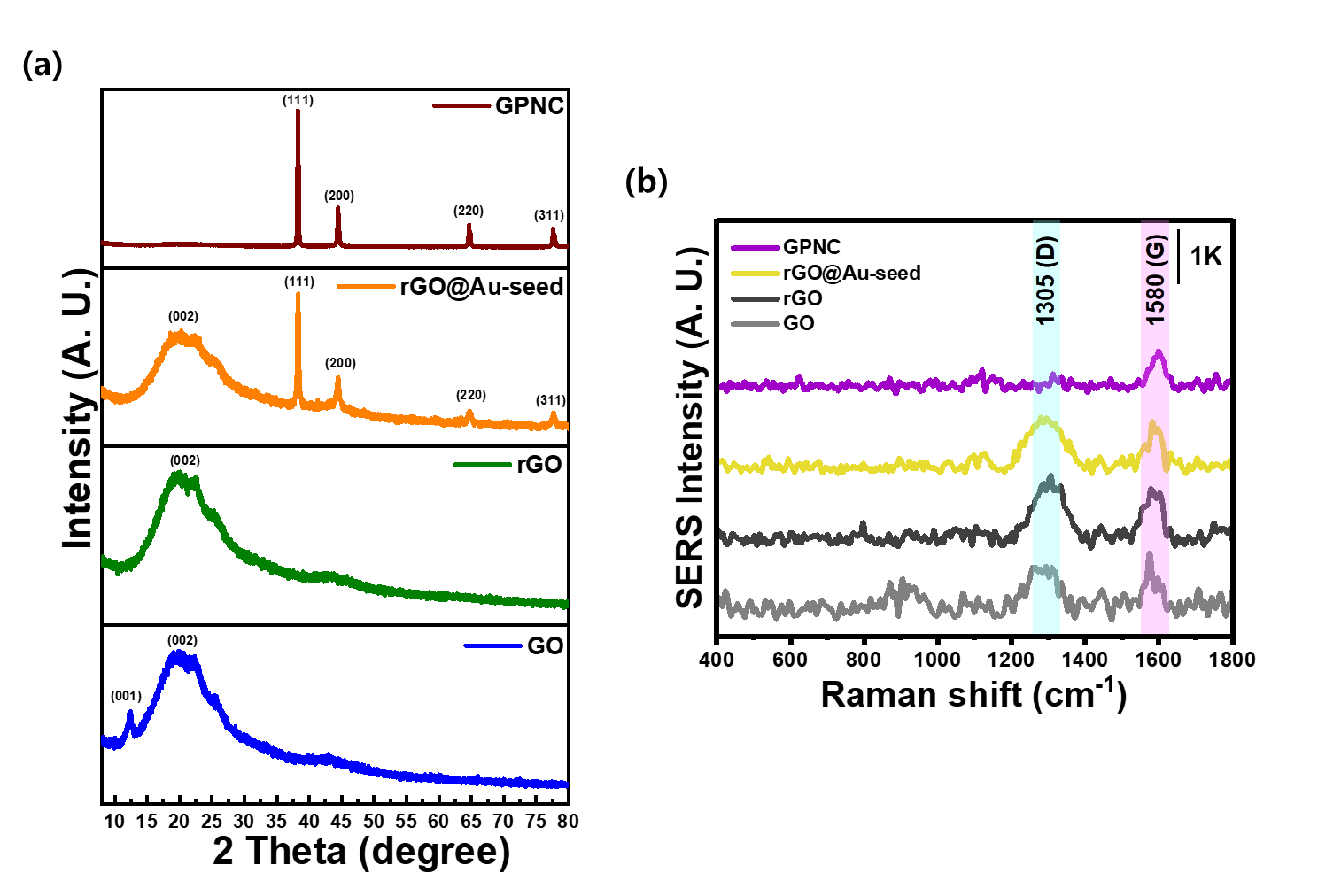
Figure S3.** (a) XRD patterns of GO, rGO, rGO@Au-seed, and GPNC substrates and their (b) Raman spectra, highlighting the graphitic D band (~1305 cm⁻¹) and G band (~1580 cm⁻¹).

**
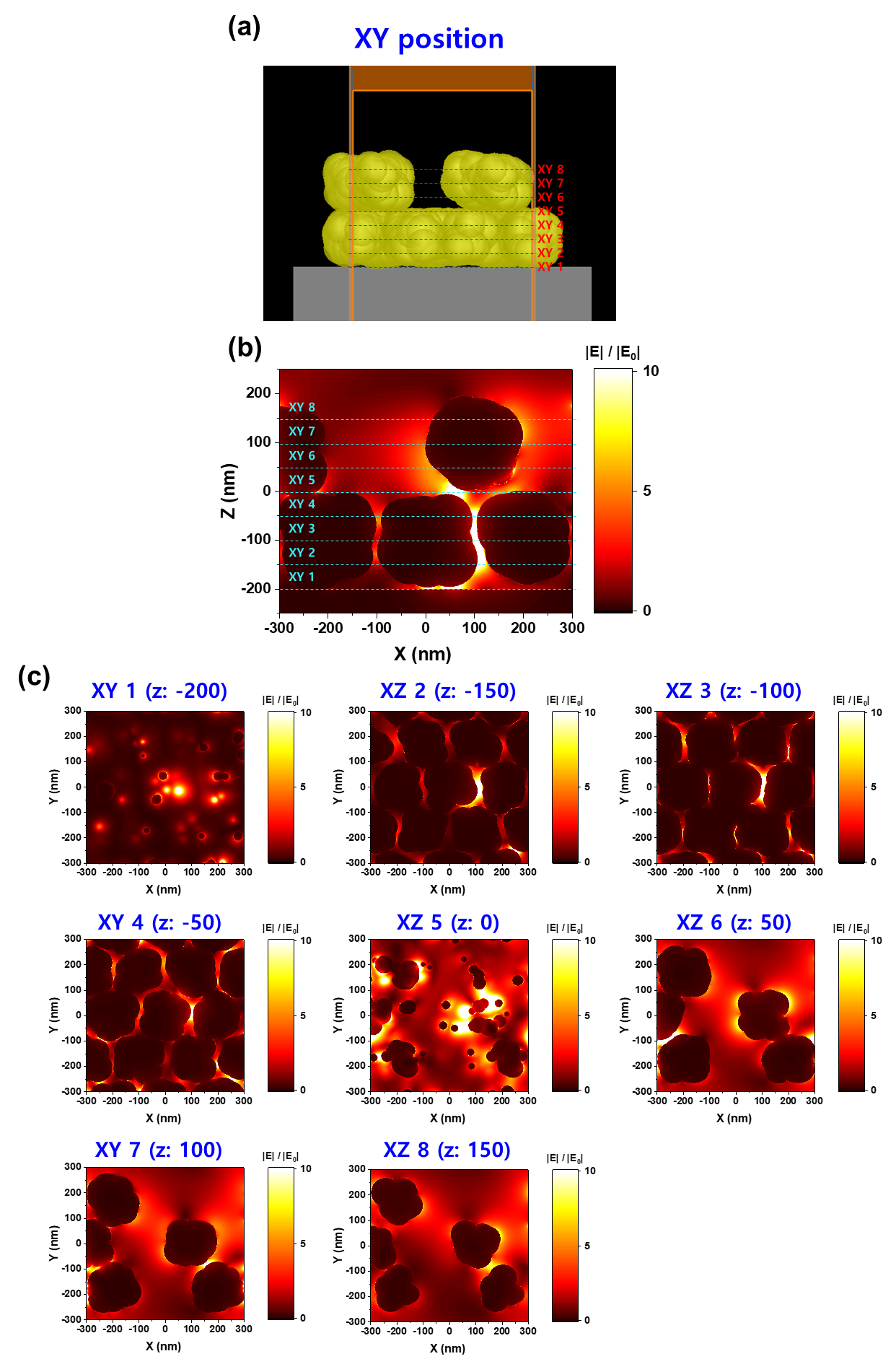
**

**Figure S4.** (a) FDTD model of the GPNC architecture, (b) simulated electric field distribution at the XZ plane, and (c) electric field distributions at multiple XY and XZ planes along the Z-axis, illustrating the evolution of the electromagnetic field across the vertical cross-section of the structure.

**
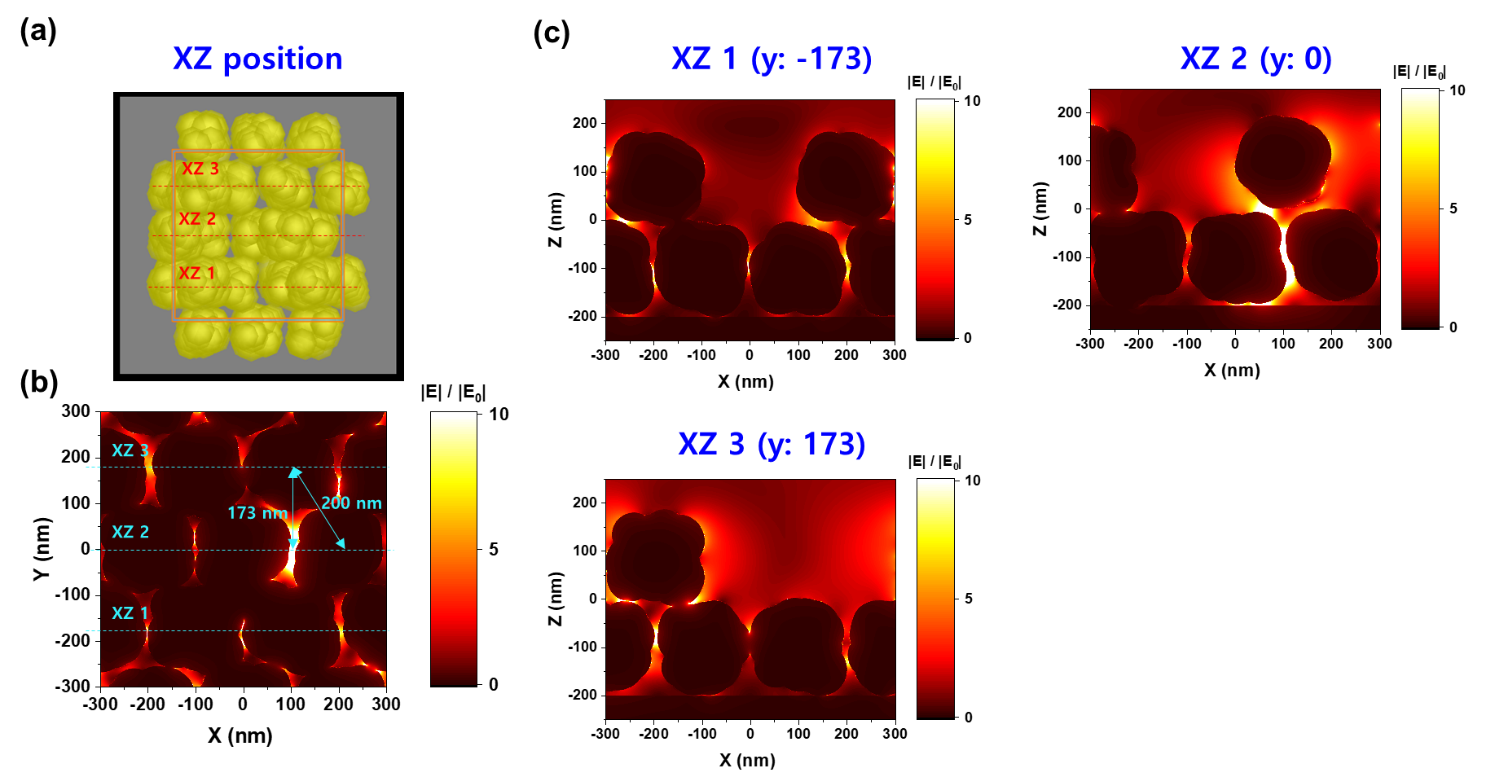
Figure S5.** (a) Schematic of the cross-sectional slicing positions in the GPNC architecture, (b) simulated electric field distribution on the central XZ plane, and (c) FDTD-simulated electric field distributions at XZ planes corresponding to y = −173, 0, and +173 nm, showing field variations along the y-axis (top-down direction).


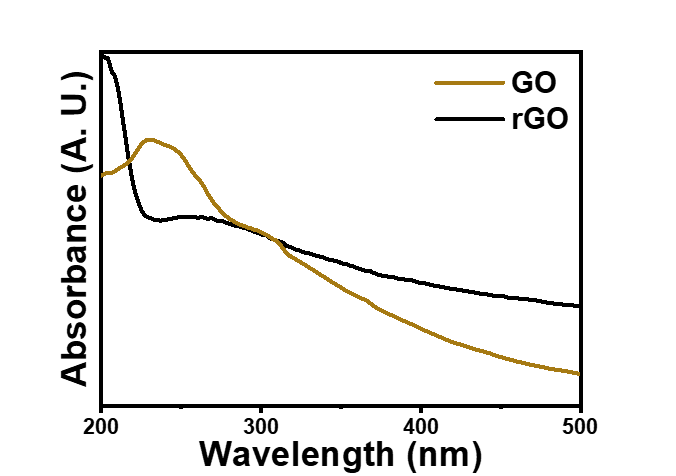


**Figure S6.** UV–vis absorption spectra of GO (brown) and rGO (black).


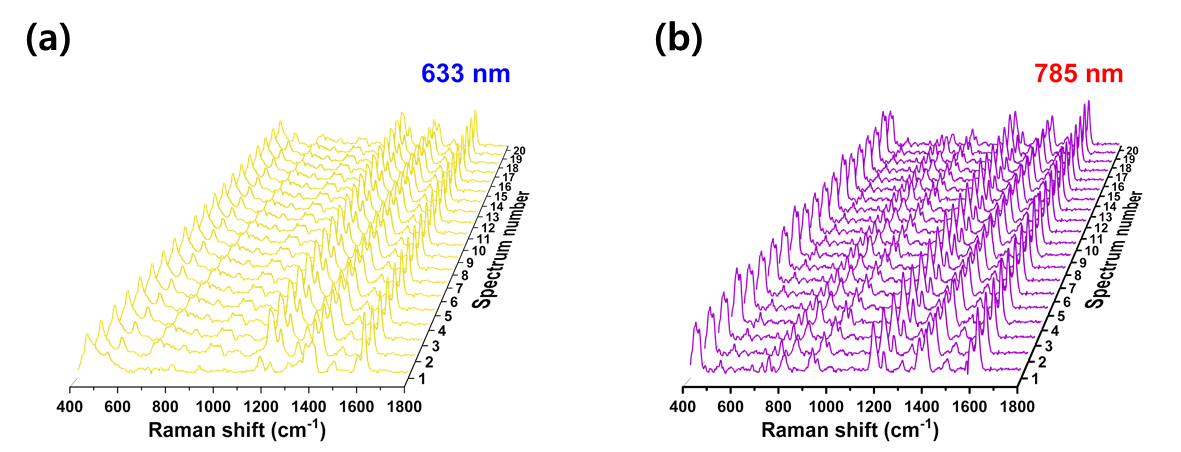


**Figure S7.** Spectral uniformity data of the GPNC substrate under (a) 633 and (b) 785 nm excitations.

**Figure S8.** Batch-to-batch reproducibility of GPNC substrates. Raman intensities of 1 µM MG were measured across three independent batches.


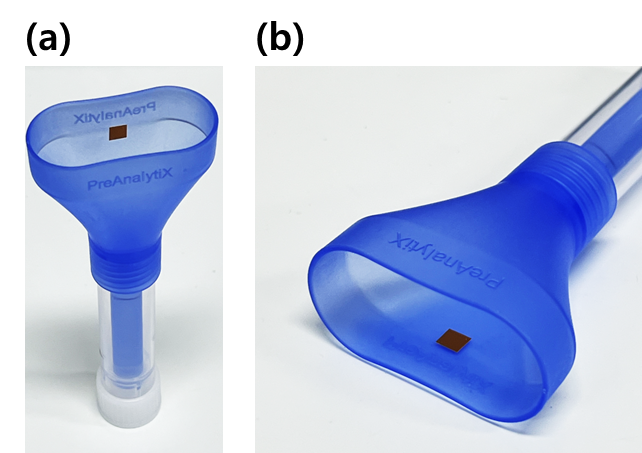


**Figure S9.** (a) Integration of the GPNC sensor into a commercial saliva collection kit, (b) angled view highlighting the sensor placement within the collection funnel.


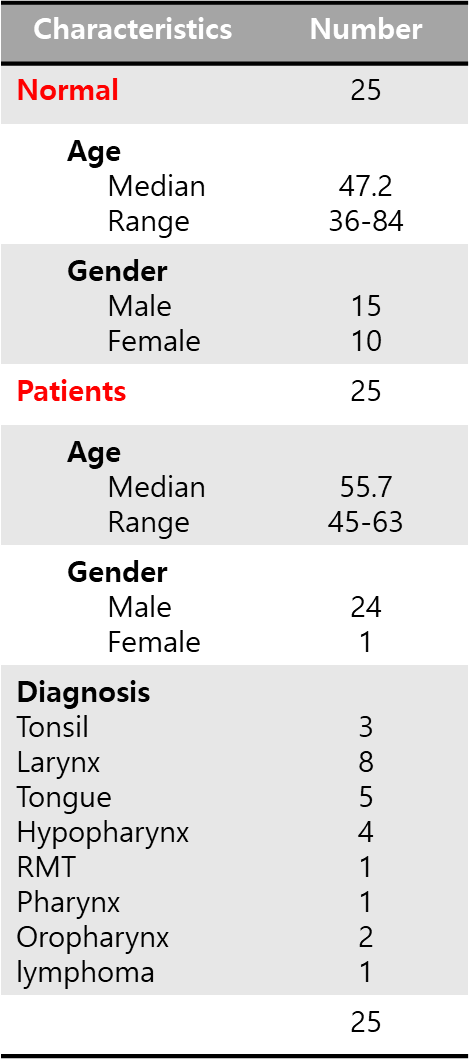


**Figure S10.** Characteristics of saliva samples collected from healthy controls (Normal) and HNC patients.


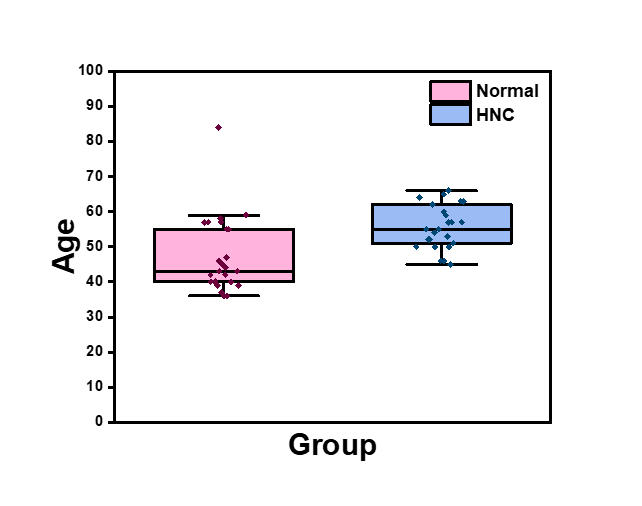


**Figure S11.** Comparison of mean age between control (Normal) and HNC groups.


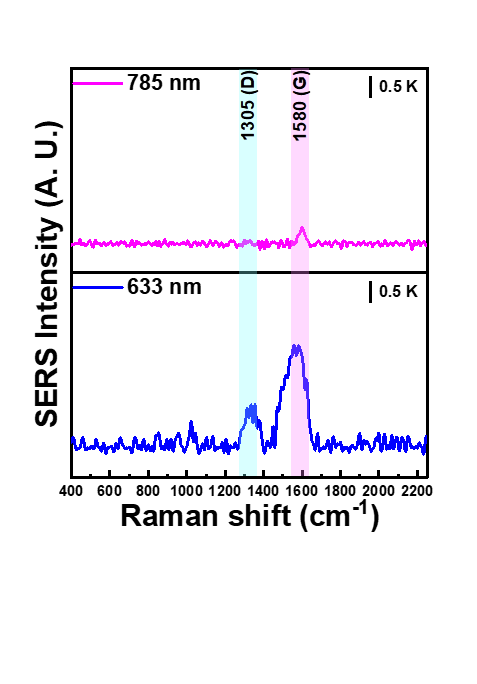


**Figure S12.** Background Raman spectra of the GPNC substrate under 633 and 785 nm laser excitation, showing differences in baseline intensity and graphitic band visibility.


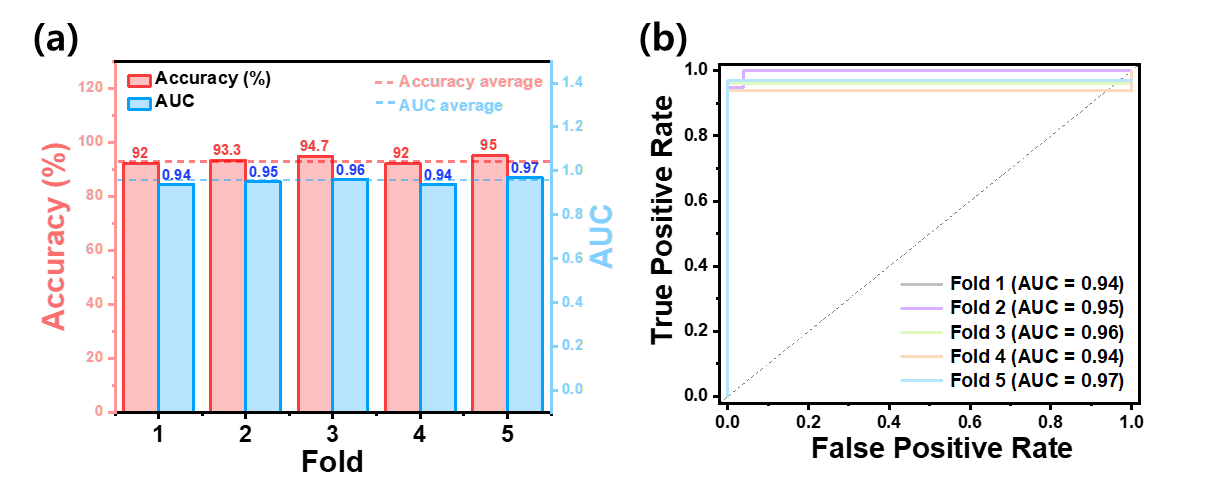


**Figure S13.** (a) Accuracy and AUC of logistic regression across 5-fold cross-validation; (b) ROC curves from 5-fold cross-validation.


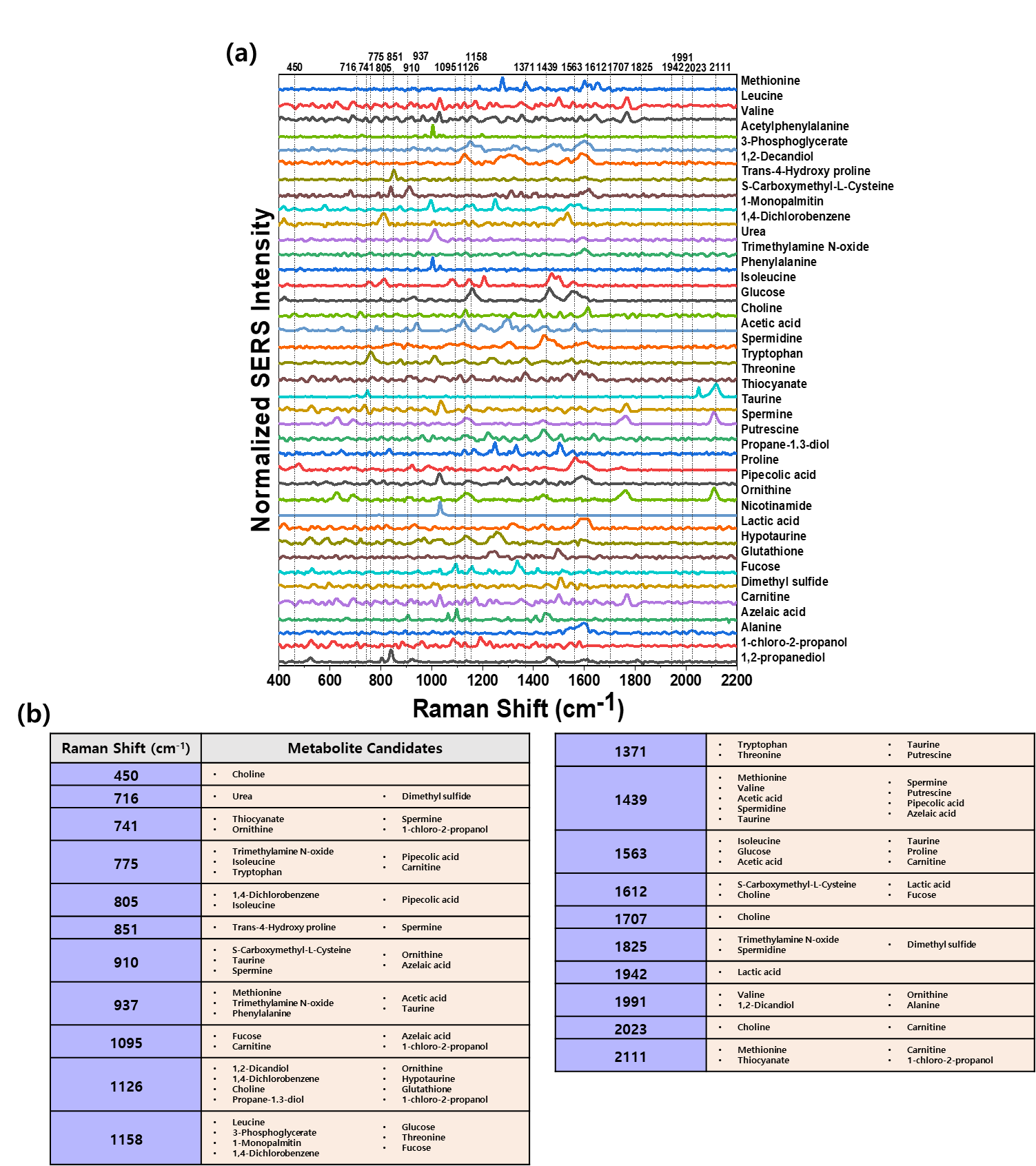


**Figure S14.** (a) SERS spectra of 39 metabolite candidates (10 mM) measured on the GPNC substrate. Dotted lines indicate peak positions corresponding to those in Figure 4d, arranged in ascending order of Raman shift, and (b) Raman spectra of metabolite candidates corresponding to the assigned Raman peaks.


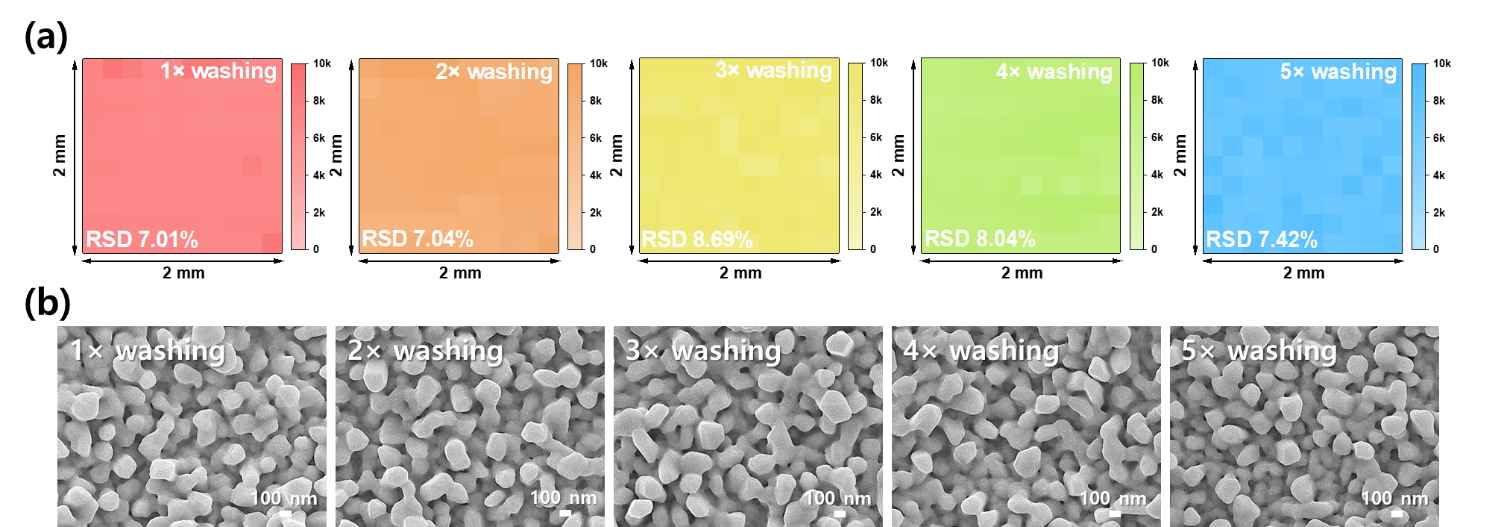


**Figure S15.** Stability of the GPNC substrate after sequential DI water washes: (a) 100-point Raman mapping results and (b) SEM images before and after five washing cycles.


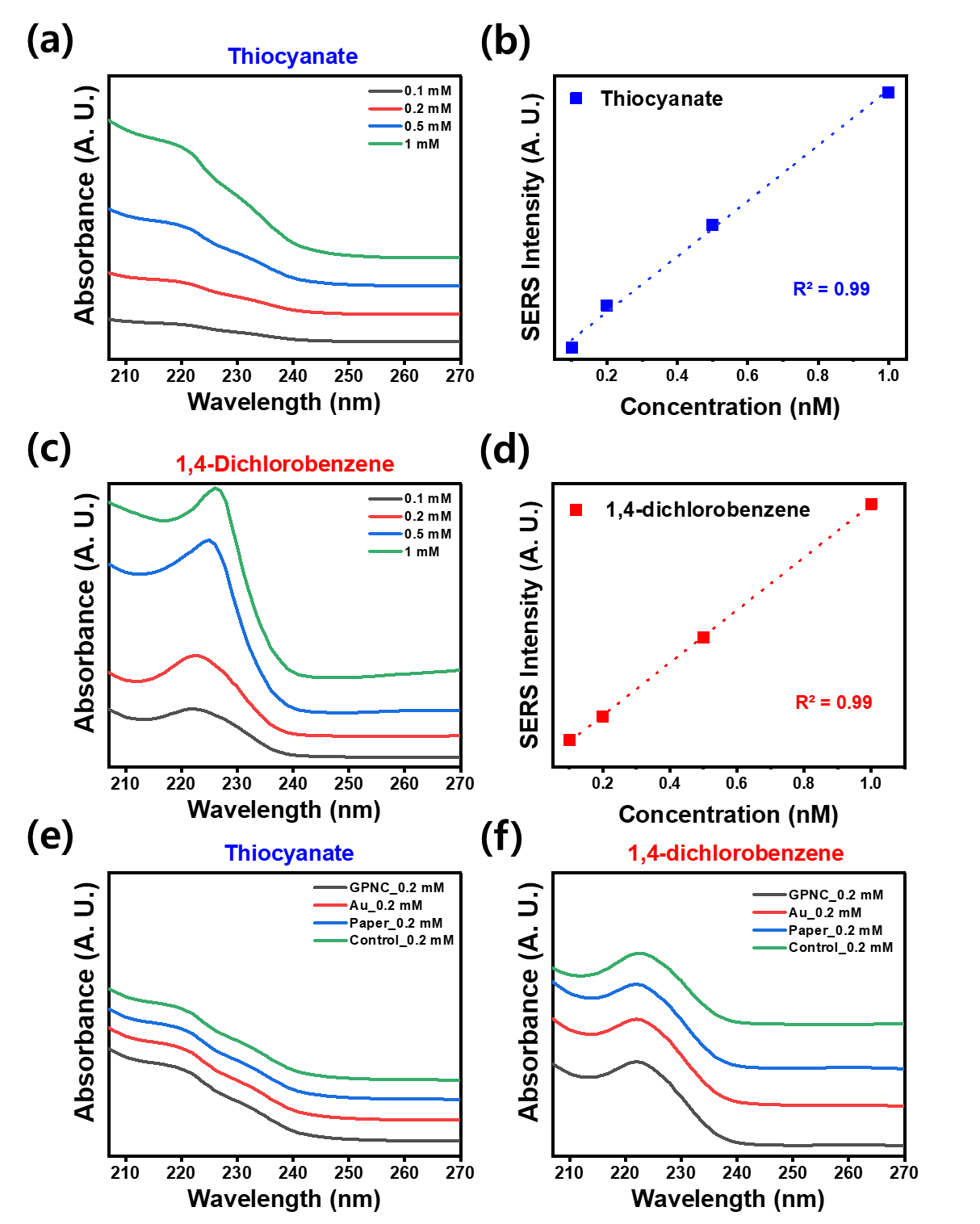


**Figure S16.** UV–vis calibration curves (a,c) and corresponding linear plots (b,d) of thiocyanate and 1,4-dichlorobenzene in the concentration range of 0.1–1 mM. UV–vis spectra of supernatants after 2 h incubation of different substrates (GPNC, Au, CA paper, control) with (e) thiocyanate and (f) 1,4-dichlorobenzene solutions at an initial concentration of 0.2 mM.


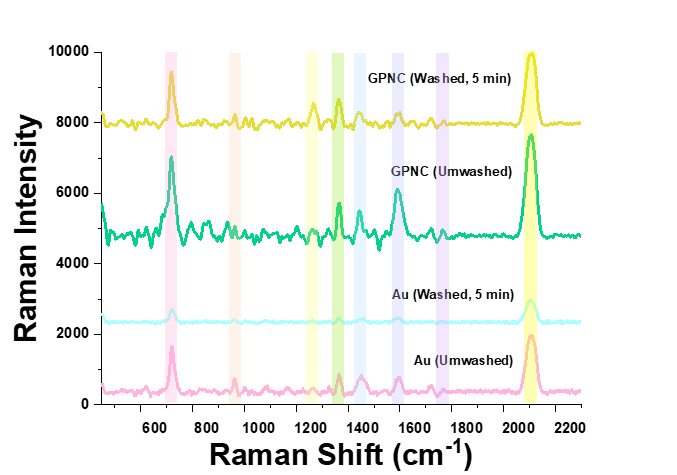


**Figure S17.** Comparison of Raman intensity reduction before and after washing on Au and GPNC substrates using a saliva sample from an HNC patient.


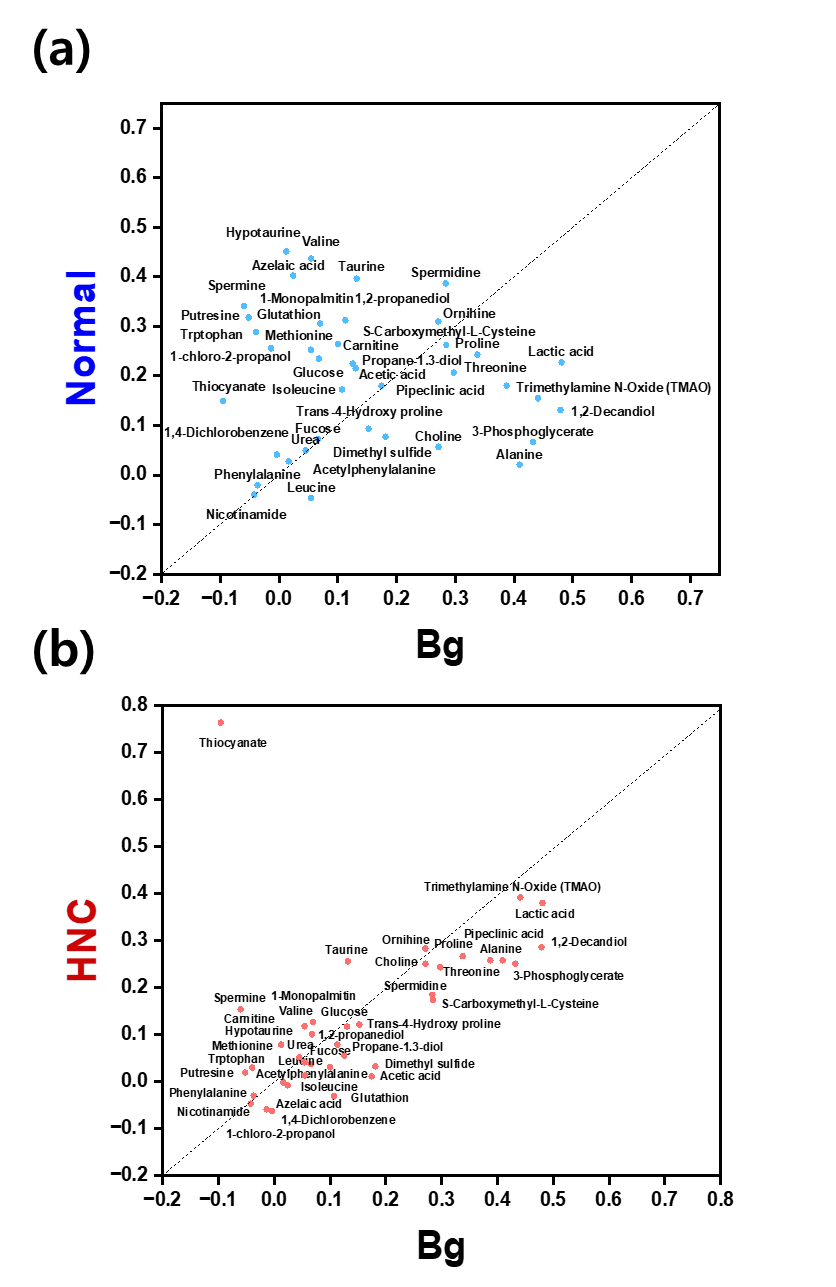


**Figure S18.** (a) PCC analysis results for potential metabolite candidates between normal and sensor background signals and (b) PCC analysis of potential metabolite candidates between HNC and sensor background signals.

| **No.** | **Metabolite** | **Up/down Regulation** | **Ref.** |
| --- | --- | --- | --- |
| 1 | Glutathione | ▲ | [40, 46] |
| 2 | 1,4-Dichlorobenzene | ▽ | [45, 48] |
| 3 | 1,2-Decanediol | ▽ | [45, 48] |
| 4 | Acetic Acid | ▲ | [45] |
| 5 | Taurine | ▲ | [45] |
| 6 | Hypotaurine | ▽ | [45] |
| 7 | Nicotinamide | ▽ | [45] |
| 8 | Lactic Acid | ▲ | [46] |
| 9 | Alanine | ▽ | [48] |
| 10 | Urea | ▽ | [48] |
| 11 | 1,2-Propanediol | ▲ | [49] |
| 12 | Fucose | ▲ | [49, 52] |
| 13 | Proline | ▽ | [49, 52] |
| 14 | Choline | ▲ | [49, 52] |
| 15 | Pipecolinic Acid | ▲ | [49, 52] |
| 16 | L-Phenylalanine | ▲ | [50] |
| 17 | S-Carboxymethyl-L-Cysteine | ▲ | [50] |
| 18 | Trans-4-Hydroxyproline | ▲ | [51] |
| 19 | Propane-1,3-Diol | ▲ | [51] |
| 20 | 3-Phosphoglycerate | ▲ | [51] |
| 21 | 1-Monopalmitin | ▲ | [51] |
| 22 | Azelaic Acid | ▲ | [51] |
| **23** | Methionine | **▲** | [52] |
| 24 | Trimethylamine N-Oxide (TMAO) | ▲ | [52] |
| 25 | Glycine | ▽ | [52] |
| 26 | Thiocyanate | ▲ | [52] |
| 27 | Spermidine | ▲ | [53, 54] |
| 28 | Spermine | ▲ | [53, 54, 55] |
| 29 | Putrescine | ▲ | [54] |
| 30 | Dimethyl sulfide | ▲ | [56, 57, 58] |
| 31 | 1-Chloro-2-propanol | ▲ | [45] |
| 32 | Glucose | ▲ | [59, 60] |
| 33 | Valine | ▽▲ | [61, 62] |
| 34 | Threonine | ▲ | [63] |
| 35 | Isoleucine | ▲ | [61] |
| 36 | Tryptophan | ▽ | [61, 64] |
| 37 | Leucine | ▽ | [61, 62] |
| 38 | Ornithine | ▲ | [48] |
| 39 | Carnitine | ▲ | [48] |

**Table S1.** Summary of reported metabolite alterations in head and neck cancer: literature evidence.
